# Comparison of group and individual therapy in physiotherapy, occupational therapy, and speech therapy: a systematic review

**DOI:** 10.3389/fresc.2026.1904321

**Published:** 2026-09-29

**Authors:** Michael Minor, Elisabeth Plath, Carsten Volland, Silke Neusser, Anna Bußmann

**Affiliations:** Essener Forschungsinstitut für Medizinmanagement (EsFoMed) GmbH, Essen, Germany

**Keywords:** group therapy, occupational therapy, physiotherapy, speech therapy, systematic review, therapeutic services

## Abstract

**Introduction:**

This review aimed to determine the effects of (digital) group therapies in comparison to individual therapies within physiotherapy, occupational therapy, and speech therapy.

**Methods:**

Embase, PubMed and Web of Science were searched until May 2025. RoB 2 and GRADE were used to rate the risk of bias and the certainty of the evidence. Mean differences were used to analyze differences between group and individual therapy.

**Results:**

Eleven studies were included in this review (eight of physiotherapy, two of speech therapy, and one of occupational therapy). The studies cover various diseases and exhibit a high degree of heterogeneity in interventions and outcomes. For 67 of the 76 outcomes, no significant differences were reported between group and individual therapy post-intervention. Group therapy showed better results in five outcomes, individual therapy showed better results in four outcomes. Three studies raised some concerns, and eight studies showed a high overall risk of bias. Certainty grading was (very) low for all outcomes.

**Discussion:**

This review found no clear evidence favoring either group or individual therapy in therapeutic services. The evidence’s derivation from heterogeneous low-quality studies with small sample sizes necessitates a careful interpretation of the results. Further research is needed to validate these findings.

## 1 Introduction

In Germany, statutory health insurance providers cover the cost for patients who are eligible for therapeutic services. In 2025, a total of 39 million therapeutic services were prescribed and 334 million treatments were performed. The three therapeutic areas with the highest number of treatments were physiotherapy (approximately 271 million treatments), occupational therapy (approximately 34 million treatments), and speech therapy (approximately 19 million treatments which cover logopedics as well as voice, speech, language, and swallowing therapy) (1). To achieve group dynamics effects and fulfill the principle of cost-effectiveness, a prescription of therapeutic services is intended for group therapy, unless individual therapy is medically necessary (2). Nevertheless, the utilization of group therapies in the context of therapeutic services remains at a very low level. In 2025, less than 0.1% of all treatments in physiotherapy, 1.6% in occupational therapy, and 0.5% in speech therapy were group treatments (1).

Group therapy in therapeutic services may be as effective as individual therapy and, in some cases, could be even more effective (3). Due to the experiences and treatment in group therapy, the perceived benefit for patients may be higher than in individual therapy (4). Since group therapy allows for the simultaneous treatment of multiple patients, it can reduce the need for personnel (5), which can improve access to therapeutic services and reduce waiting times (6). Group therapy can therefore be more cost-effective than individual therapy (7). Despite the advantages group therapy can offer, there are also disadvantages to these treatment methods. Not all studies on group therapy consistently yield the same health-related outcomes as individual therapy. In their study comparing pelvic floor training in individual and group therapy, Bech et al. concluded that the effect on pelvic floor strength was higher in individualized therapy than in group therapy (8). In addition, group therapy sessions may have problems with patients' regular attendance (9). Also, before a group therapy session can begin, it is essential to first recruit a sufficient number of patients within a designated time frame (10).

Besides that, the German healthcare system allows for therapeutic services to be delivered as a telemedicine service (2). The effectiveness of telemedicine in therapeutic services can be equivalent to that of in-person care (11). Moreover, telemedicine can increase the satisfaction of therapists and patients (12), as well as improve access to healthcare. This is particularly beneficial for patients who live in rural areas or have limited local treatment options (13). Although digital therapies are permitted, the German Federal Joint Committee, which is responsible for the German directive on therapeutic services, has expressed concerns regarding the delivery of therapeutic services as telemedicine services. Accordingly, telemedicine may result in limited auditory, tactile and visual perception. Because not the same observations, impressions and findings can be gathered as in in-person care, there is a risk that therapists might not be able to fully understand the patients' complaints and their personal and environmental background factors (14).

Currently, there is no comprehensive evidence that compares the effects of (digital) group therapies to individual therapies using identical interventions in physiotherapy, occupational therapy, and speech therapy. It remains uncertain whether (digital) group therapies are appropriate for these therapeutic services. Considering individual and group therapy from an indication-independent perspective within therapeutic services is essential, as it highlights the overall effectiveness that transcends specific diagnoses. This approach facilitates the creation of transferable evidence that can inform the provision of therapeutic services. By including physiotherapy, occupational therapy, and speech therapy, it is possible to investigate whether similar effects and outcomes can be identified across different therapeutic services. Therefore, this systematic review aims to empirically evaluate the effects of (digital) group therapies compared to individual therapies in the field of physiotherapy, occupational therapy and speech therapy. Furthermore, this review is also intended to provide further insight into which indications are potentially suitable for (digital) group therapy.

## 2 Methods

The methodology of the systematic review is based on the Cochrane Handbook for Systematic Reviews of Interventions (15). The Preferred Reporting Items for Systematic Reviews and Meta-Analyses (PRISMA) checklist is used for the approach and presentation (16). The PRISMA checklist is found in Supplementary Material 1.

### 2.1 Eligibility criteria

This systematic review includes studies in which the effects of group therapies or digital group therapies were compared to individual therapies or digital individual therapies in physiotherapy, occupational therapy, or speech therapy. Studies were included only if group and individual interventions were delivered in the same format (both in-person or both digital). If interventions were conducted digitally, inclusion required synchronous communication in the form of an online treatment via video in real time between therapists and patients, according to the German healthcare system (17–19), whereas the mode of outcome assessment (in-person or online) was not used as an inclusion criterion. To evaluate the differences in the effects of group and individual therapy, only studies were included in which the interventions in group and individual therapy were based on the same therapy protocol and were thus comparable. Since, in the evaluation of intervention effects, biases in non-randomized studies can be higher than in randomized studies, and non-randomized studies differ in their suitability for estimating causal effects (20), only randomized controlled trials (RCTs) were included in the systematic review. Furthermore, in addition to randomization of participants, a comparison had to be made between an intervention (group therapy) and a control group (individual therapy). Studies were included if they were written in German or English and if abstracts and full texts were available.

Studies of other therapeutic service areas and psychotherapeutic interventions were excluded. Studies that do not define the effects of (digital) group therapies as an outcome were not included. Additionally, studies in which the interventions differed between group and individual therapy were excluded. Studies without a comparison between group and individual therapies were also excluded. Digital interventions without synchronous communication between therapists and patients, such as recorded videos or digital health applications, were not considered, as they are not regarded as telemedicine services according to German therapeutic services (2). The exclusion of self-management interventions and educational measures without the guidance of therapists was equally consistent.

To ensure a comprehensive view of the effects of group therapy on healthcare provision, no restrictions were imposed based on specific demographic (e.g., age, gender) or health characteristics. Similarly, no restrictions were imposed regarding publication date, study location, or sample size.

### 2.2 Information sources and search strategy

To identify potentially relevant articles, a search was conducted in Embase, PubMed, and Web of Science in May 2025. In accordance with the Cochrane Handbook for Systematic Reviews of Interventions, the population, intervention, comparison, and outcome (PICO) framework was used to develop the search strategy (21). The PICO framework divides the search strategy into four key components:

- population: patients receiving physiotherapy, occupational therapy or speech therapy
- intervention: group therapy (in-person or digital)
- comparison: individual therapy (in-person or digital)
- outcome: effects/effectiveness/indications

The search strategy aims to ensure a high level of sensitivity and avoid excluding relevant studies by focusing the search too narrowly. Therefore, only the components “population” and “intervention” were operationalized for the development of the search string. The “population” component was defined with the relevant terminology for the three therapeutic services physiotherapy, occupational therapy, and speech therapy, ensuring a comprehensive and precise capture of the pertinent studies. Additionally, the “intervention” (group therapy) was operationalized because it represents the core treatment modality to be investigated as the research question focuses on the intervention format. Specifying comparison parameters or outcomes would have limited the search and potentially excluded relevant studies. To minimize this risk of over-restriction, “comparison” and “outcome” were not included in the search strategy but were instead manually screened during the title and abstract screening. This allowed to include studies only when they met both the intervention-format requirement and the predefined scope of therapeutic service. Therefore, the search was intentionally restricted to group-format terminology and therapeutic services terms, rather than diagnostic or demographic keywords. Search terms and synonyms for the components “population” and “intervention” were identified and expanded using thesauri from the searched databases, such as Medical Subject Headings and Emtree.

To create the search string, the search terms and components were combined using Boolean operators. To obtain a robust evidence base with high accuracy and completeness for the systematic review, the search strategy was validated according to the Peer Review of Electronic Search Strategies guideline (22). The literature search was conducted using both English and German terms to ensure a more comprehensive coverage of the relevant evidence. The generic search string, as shown in Table 1, was adapted to the specific features of the various databases. The complete database-specific search strings, including the number of hits, are shown in Supplementary Material 2.

**Table 1:** Search string.

Components	Search terms
Population: physiotherapy	“physiotherap*” OR “physical therap*” OR “exercise therap*” OR “manual therap*” OR “physiatric*”
Population: occupational therapy	“activit* of daily living” OR “occupational therap*” OR “ADL” OR “ergotherap*”
Population: speech therapy	“speech therap*” OR “language therap*” OR “voice therap*” OR “logop*” OR “voice training” OR “myofunctional therap*” OR “swallowing therap*” OR “speech correction”
Intervention: group therapy	“group-based” OR “group therap*” OR “group session*” OR “group intervention*” OR “group treatment*” OR “group setting*”

### 2.3 Selection and data collection process

The search results were exported to EndNote and duplicates were removed. First, all titles and abstracts were reviewed. The selection of studies was based on the inclusion and exclusion criteria. In addition, the primary studies identified in relevant systematic reviews through the literature search were assessed for their suitability for inclusion. Based on this, relevant full texts were selected, obtained, and reviewed. The screening of titles, abstracts, and full texts was conducted independently by two researchers (EP and MM). In case of discrepancies, a further member of the study team was consulted to reach a consensus. No automation tools were used in the selection process, all steps were performed manually. References that did not meet the inclusion criteria were excluded. With the initiation of full-text screening, the reasons for exclusion were documented.

To determine which data should be extracted, the research team developed a data extraction table. After reviewing the first studies, the table was revised in an iterative process. The data extraction was carried out independently by two researchers (EP and MM) in Microsoft Excel. The extracted data were compared, and any discrepancies were clarified through discussion. The following data were systematically extracted for all included studies: general study data (author, year, title, country), study type and design (randomization, presence of intervention and control group, data collection period, setting), study population (inclusion and exclusion criteria, number of included and treated patients, dropout rate), intervention and control (therapeutic service area, duration, description of group and individual therapy, group size), and outcomes (endpoints, data collection instruments, measurement time points, effect measures). Since the included studies used different therapeutic interventions, target groups, measurement instruments, and endpoints, the extraction was kept open in terms of content. The focus was particularly on describing the reported effects. The extraction table with the data from all included studies is provided in Supplementary Material 3.

### 2.4 Study risk of bias assessment

The risk of bias assessment of the included studies was performed using the Cochrane risk-of-bias tool for randomized trials (RoB 2). This tool is recommended for randomized studies (23). RoB 2 is divided into five different domains that focus on different aspects of the study design, study conduct, and reporting of results. These domains address all the major risks of bias that can influence the results of randomized studies. The evaluation of the risk of bias is determined by the three categories “low risk”, “some concerns”, and “high risk”. The overall risk of bias is determined by the evaluations of individual domains and is represented by the lowest rating among those domains (24). The risk of bias was evaluated using the templates for completion (25) by two researchers (EP and MM) independently. Any discrepancies in the evaluations were discussed between the two researchers to reach a consensus.

### 2.5 Effect measures

Due to the heterogeneity of the included studies, a quantitative meta-analysis was not conducted. The studies differed substantially with regard to clinical characteristics, methodological features, interventions, and outcomes. Clinical heterogeneity resulted from differences in patient populations, and diagnoses. Methodological heterogeneity included variation in study designs, sample sizes, intervention settings, treatment frequency, duration, and delivery format. In addition, outcome-related heterogeneity was present because studies assessed a wide range of endpoints using different and often non-compatible measurement instruments. As a result, the reported effect estimates were not sufficiently comparable to justify statistical pooling.

Continuous outcomes were summarized descriptively using the effect measures reported in the individual studies, such as mean differences, to depict continuous variables in each study. Statistically significant between-group differences (*p* < 0.05) between individual and group therapy were highlighted where reported by the original studies. Non-metric outcomes, such as satisfaction, were summarized narratively. For the qualitative synthesis, the reported effects of the comparisons between individual and group therapy were categorized as “no difference”, “favors group therapy”, or “favors individual therapy”.

### 2.6 Synthesis methods

After the data were extracted in tabular form, similar outcomes from the studies were sought. To ensure consistent representation, comparable outcomes were grouped into domains and subsequently described narratively. The results were thematically structured into the following categories: “motor function, physical performance, and physiological parameters”, “voice-related outcomes”, “adaptive behavior and activities of daily living”, “pain”, “quality of life and psycho-social factors”, and “attendance, compliance, satisfaction, and process indicators”.

### 2.7 Certainty assessment

To evaluate the certainty of evidence for the outcome domains, the Grading of Recommendations Assessment, Development, and Evaluation (GRADE) tool, as recommended by Cochrane, has been used (26). GRADE offers a structured framework to classify evidence into four categories (high, moderate, low, and very low) based on its certainty. In intervention studies, certainty refers to the effects of the treatment. The starting point for rating the certainty of evidence for interventions is the study design. RCTs, as included in this review, start with a high-certainty rating. This first rating is followed by more detailed ratings using five different criteria that can lower the certainty of the evidence. These judgments provide a final determination of the certainty of the evidence for each outcome domain (27).

## 3 Results

A total of 7,005 records were identified through the systematic literature search in the databases. After removing 2,530 duplicates, 4,475 records were screened. Based on their titles and abstracts, 4,397 records were excluded. Since two reports could not be retrieved, 76 full texts were assessed for eligibility in accordance with the established inclusion and exclusion criteria. This resulted in the exclusion of 67 full texts: in 36 studies, the interventions between group and individual therapy were not comparable; in 27 studies, no randomization was performed; in three studies, interventions and analyses of group and individual therapies were mixed; and in one study, no group therapy was conducted. Nine studies (28–36) from the systematic literature search met all inclusion criteria and could be included in this review.

Additionally, two further studies (37, 38) were found, resulting in a total of eleven studies included in this review. During the title and abstract screening, other reviews were identified that had thematic similarities to the contents of this review. The selected reviews were thoroughly examined, and the titles and abstracts of all the studies included were screened for the inclusion and exclusion criteria of this review. Eight studies were identified, and their full texts were assessed for eligibility. Five studies were excluded because the interventions between group and individual therapy were not comparable, as well as one study in which no randomization had occurred. The PRISMA flow diagram of the screening process is shown in Figure 1. The full-text screening and reasons for exclusion are listed in Supplementary Material 4.

**Figure 1:**
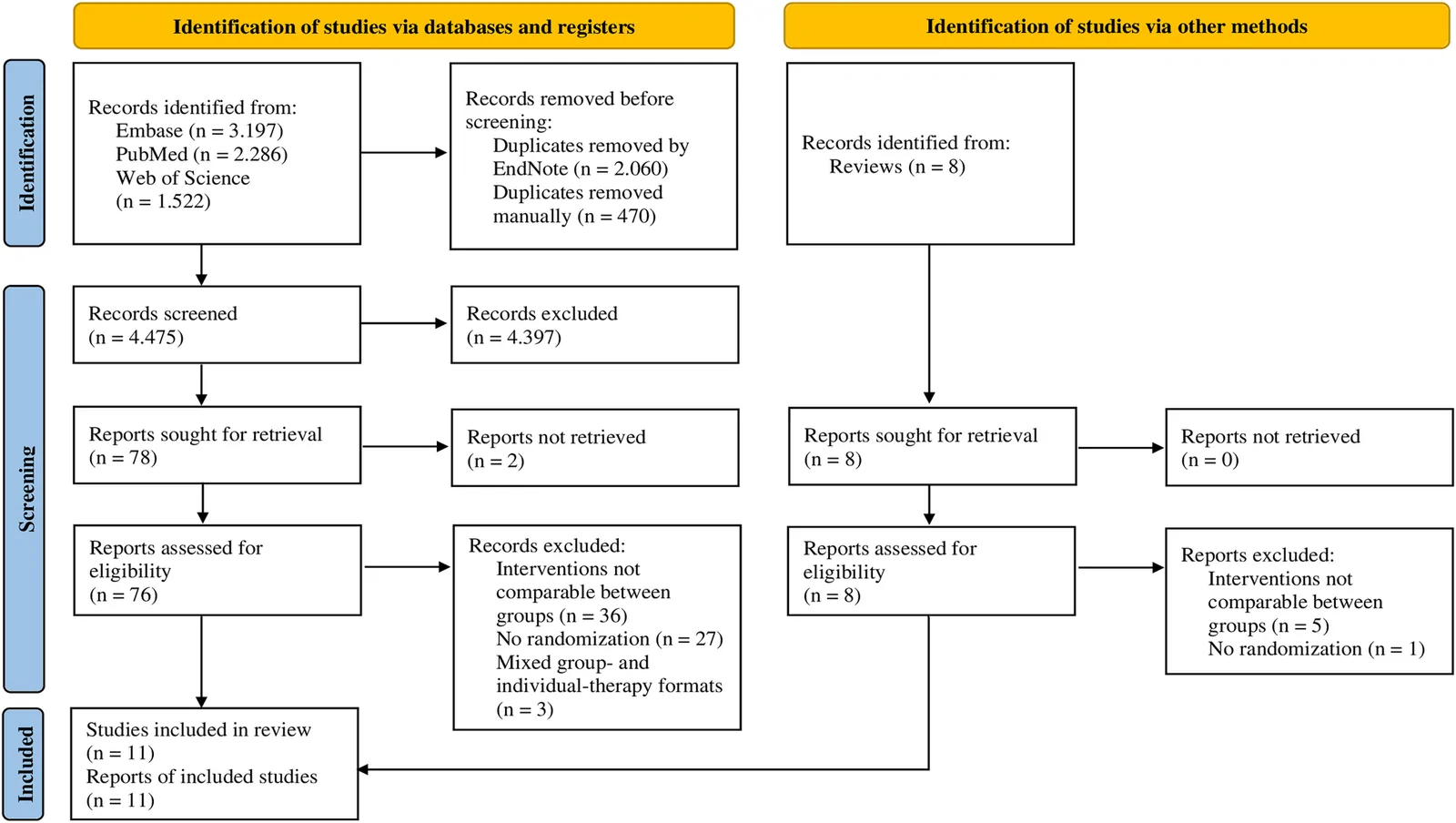
PRISMA flow diagram.

### 3.1 Study characteristics

The included studies cover a wide range of interventions from the three therapeutic service areas. They are characterized by a high degree of heterogeneity in planning and implementation. Eight studies are related to physiotherapy (28–30, 32, 34, 35, 37, 38), two to speech therapy (33, 36), and one to occupational therapy (31). All included studies were conducted in-person, none of the interventions were carried out as digital therapy.

In the field of physiotherapy, three studies examined the treatment of patients with paralysis after a stroke using constraint-induced movement therapy (30), task-oriented circuit training (38) or mirror therapy (35). One study evaluated the effects of exercise training on the motor performance of children with developmental coordination disorders (32). Pereira et al. conducted pelvic floor muscle training for females with stress urinary incontinence (34). Two studies evaluated a multimodal physiotherapy program for patients with Parkinson's disease (28) or chronic knee pain (37). One study examined strength training for resistance trained males (29).

Karalı et al. investigated the effects of voice therapy in pediatric professional voice users with vocal fold nodules (33). In the study by Zamani et al., children who received cochlear implants received a narrative-based language intervention (36).

The study in the field of occupational therapy was conducted with toddlers with autism. Participants received therapy based on the developmental, individual-differences, and relationship-based floortime model (31).

The therapies were conducted in ambulatory or clinical settings. The number of patients treated ranged from small pilot studies with 13 patients to larger studies with 229 patients. Except for the largest study, all other studies included in this review were relatively small, with the second-largest study enrolling 60 patients, while the remaining studies included even fewer. Depending on the study, the number of participants in the group therapies ranged from a minimum of two to a maximum of ten people, with group sizes fluctuating even within the same study. The shortest intervention lasted two weeks, and the longest lasted 12 weeks. In each study, between six and 24 sessions were conducted. Depending on the study, a single session could last between 30 and 180 min. In some studies, one, two, or three sessions were conducted per week, and in one study, one session was conducted daily. Most studies measured outcomes before and after the intervention. Only three studies conducted follow-ups after two or six months. A complete overview of the study characteristics is presented in Supplementary Material 5.

### 3.2 Risk of bias in studies

Table 2 provides an overview of the risk of bias assessment for all included studies. Eight out of eleven studies showed a high overall risk of bias. The remaining three studies raise some concerns in the overall risk of bias assessment. None of the studies showed a low risk of bias across all domains.

**Table 2:** Risk of bias domain-level judgements for each individual result.

Study	Risk of bias domains					
	D1^1^	D2^2^	D3^3^	D4^4^	D5^5^	Overall
Ciortea et al. (28)	−	x	x	+	−	x
Dorrell et al. (29)	−	+	+	+	−	−
Doussoulin et al. (30)	x	x	+	+	−	x
Hirschmann et al. (31)	+	x	+	+	−	x
Hung et al. (32)	+	+	+	+	−	−
Hurley et al. (37)	+	−	+	+	−	−
Karalı et al. (33)	−	x	x	+	−	x
Pereira et al. (34)	+	x	+	+	−	x
Song et al. (38)	−	x	x	+	−	x
Thieme et al. (35)	+	+	x	+	−	x
Zamani et al. (36)	+	+	x	+	−	x

**Domains.**

1 Bias arising from the randomization process.

2 Bias due to deviations from intended interventions.

3 Bias due to missing outcome data.

4 Bias in measurement of the outcome.

5 Bias in selection of the reported result.

**Judgement.**

x High risk.

− Some concerns.

+ Low risk.

Upon examining the individual domains (Figure 2), it was found that there are high risks of bias, particularly concerning deviations from the intended interventions and missing outcome data. However, assessments of high risk are often based on the absence of relevant information and not necessarily on a clearly inadequate study methodology. This also applies to domain 5 (bias due to selection of the reported results), where all studies raise some concerns. This is a typical problem in publication practice (39), as no pre-registered study protocols or published pre-specified analysis plans were available for the studies included in this review, which limits the transparency of the analyses.

**Figure 2:**
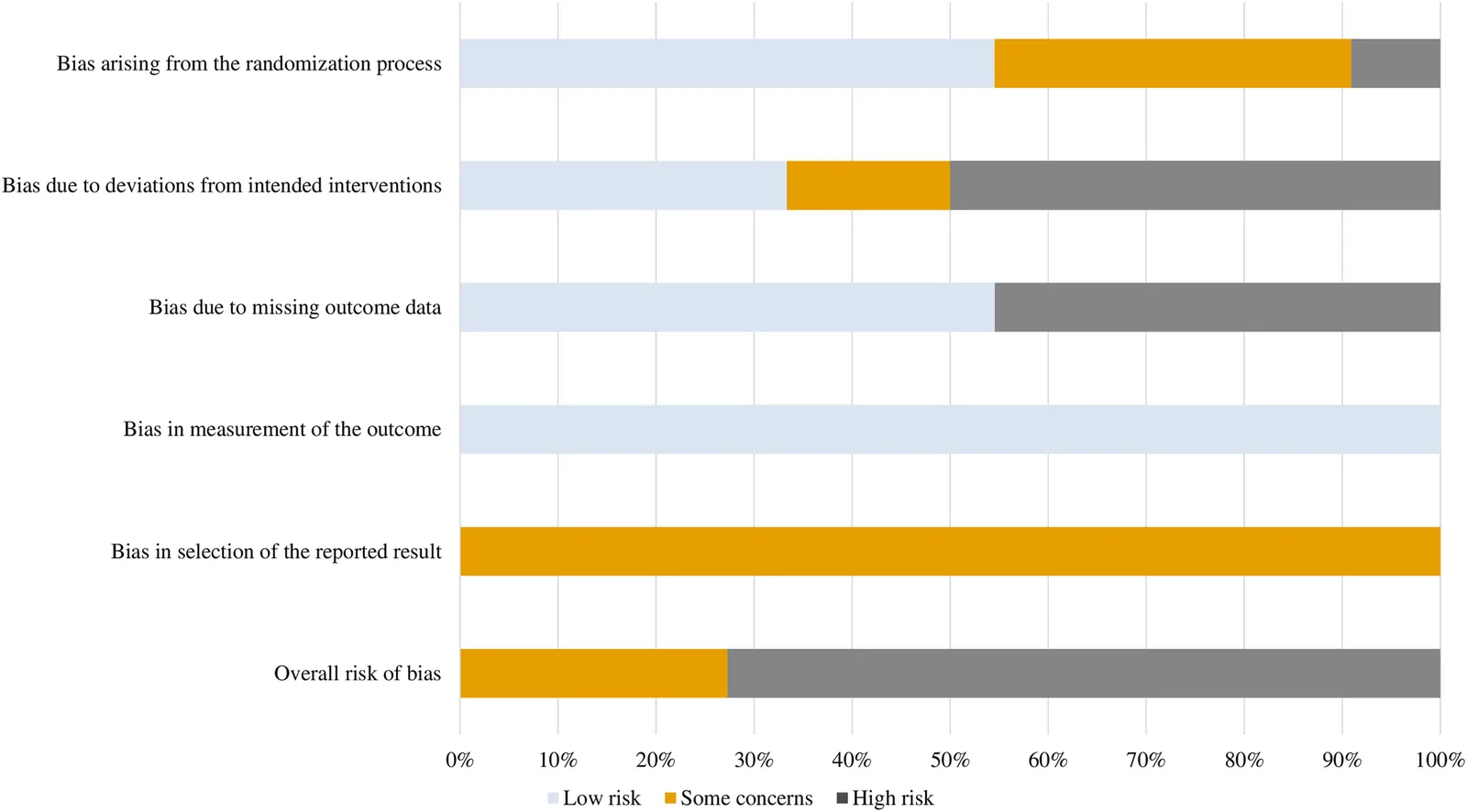
Distribution of risk of bias judgements within each bias domain.

The overall evaluation shows that the included studies have methodological weaknesses. These can potentially lead to an over- or underestimation of the actual effects, thereby not excluding the possibility of bias effects. A positive aspect is the low risk of bias due to the measurement of the outcome of all the included studies. On the one hand, the measurement instruments have a high validity. On the other hand, the same methods were used to measure outcomes because the interventions used in the individual and group therapy were based on identical intervention protocols. Although blinding of participants and therapists is not possible in comparison of group and individual therapy due to the study design, the lack of blinding in the current assessment of objective endpoints is considered significantly less problematic than in the case of subjective endpoints (40).

### 3.3 Results of individual studies

A total of 76 different outcomes with a variety of different instruments for data collection were evaluated in the eleven included studies. Supplementary Material 6 provides a complete overview of all data collection instruments, outcomes, and a comparison of outcomes between individual and group therapy per study, measured after the intervention was conducted (posttest). The results of the studies are grouped by outcome domains and presented for comparison between individual and group therapies.

### 3.4 Results of syntheses

#### 3.4.1 Motor function, physical performance and physiological parameters

Nine studies, including 533 patients, evaluated outcomes related to motor function, physical performance, and physiological parameters (28–32, 34, 35, 37, 38). Six of nine studies found no significant differences in outcomes between individual and group therapy regarding motor function, physical performance, and physiological parameters (29, 31, 32, 34, 37, 38). One study evaluated fine motor skills using the Peabody Developmental Motor Scales Second Edition (31). An additional study used the Movement Assessment Battery for Children to evaluate motor performance (total impairment score, manual dexterity, ball skills and static/dynamic balance) (32). Another study examined physical function using the Likert version of the Western Ontario and McMaster Universities Osteoarthritis Index, as well as quadriceps maximum voluntary contraction and voluntary activation (37). Furthermore, one study evaluated urinary loss using a pad test, pelvic floor muscle pressure using a Perina Stim device, and pelvic floor muscle strength by digital palpation (34). GAITRite was used in one study to test gait abilities (velocity, cadence, stance and swing phase symmetry profile, step length symmetry profile, two-minute walking test) in patients (38). Comparable outcomes were observed in all six studies following the implementation of the individual and group therapy, with no statistically relevant difference between the groups. Nevertheless, in one of these studies, in which strength assessments were compared using a back squat one-repetition maximum and jump assessments using countermovement, static-squat, and standing-broad jumps, the individual therapy caused larger percentage increases in all areas of assessment compared to group training, even though the results were not significant (29).

The results of the three other studies (28, 30, 35) that measured outcomes related to motor function, physical performance, and physiological parameters differ partially for individual and group therapy. Differences in improvements between the therapy groups in favor of group therapy were measured in two studies (28, 30). One study evaluated gait speed based on 6-minute and 10-meter walk tests. Although there was an increase in gait speed in both groups after treatment, significantly higher values were shown in the group therapy compared to the individual therapy by the 6-minute walk test scores (28). The Chilean versions of the Motor Activity Log−30 (for amount of use/quality of movement) and the Action Research Arm Test (for motor function) were used to evaluate differences between individual and group therapy in one study. Both scores increased for the group and for the individual therapy, with the increases being higher in the group therapy (30). In contrast, one study measured improvements in a single outcome in individual therapy compared to group therapy. This study evaluated arm function on an impairment level, somatosensory, and range of motion sections with the Fugl-Meyer Test, arm function on a functional level with the Action Research Arm Test, changes in resistance to passive movement of finger and wrist flexors with the Ashworth Scale, and visuospatial neglect with the Star Cancellation Test. No significant group differences were found except in resistance to passive movement of finger flexor, which indicated higher scores for individual therapy than group therapy after the intervention (35). Based on low-level evidence (GRADE) because of a very serious risk of bias in the studies, observed outcomes for motor function, physical performance and physiological parameters did not show clear differences between group and individual therapy.

#### 3.4.2 Voice-related outcomes

Voice-related outcomes were evaluated in two studies including 40 patients (33, 36). One study analyzed the s/z ratio and maximum phonation time, as well as acoustic parameters such as F0, shimmer, jitter, and the noise harmonic ratio, using different speech and voice diagnostic procedures (/i/,/s/, and/z/ phonation, and recordings). Those who participated in individual therapy had a higher s/z ratio, but group therapy participants had better noise harmonic ratio scores. In the other parameters, no significant differences were found (33). The other study examined the productivity, complexity, accuracy, and story grammar of spoken narratives. To assess storytelling abilities, the Persian version of the Language Sample Analysis test was administered. Measurements showed no differences between the groups. Both individual and group therapy led to similar improvements in all examined outcomes (36). Based on very low-level evidence (GRADE) because of a very serious risk of bias and small sample sizes in the studies, observed outcomes for voice-related outcomes did not show clear differences between group and individual therapy.

#### 3.4.3 Adaptive behavior and activities of daily living

The evaluation of adaptive behavior and activities of daily living was conducted in three studies including 341 patients (31, 35, 37). The assessment of adaptive behavior (concept, social, practical and total score composite) was conducted through the utilization of the Adaptive Behavior Assessment System questionnaire in one study. The study reported no significant differences between the adaptive behaviors in group therapy and those in individual therapy at the end of the study (31). One study evaluated objective functional performance based on the time taken to perform four common activities of daily living. No statistically relevant group differences were found (37). The evaluation of basic activities of daily living was conducted using the Barthel Index. A comparison of individual and group therapy revealed no differences in the extent of improvements observed in activities of daily living. The outcomes of both therapeutic modalities demonstrated comparable results (35). Based on very low-level evidence (GRADE) because of a very serious risk of bias and small sample sizes in the studies, observed outcomes for adaptive behavior and activities of daily living did not show clear differences between group and individual therapy.

#### 3.4.4 Pain

Two studies, including 317 patients, evaluated pain (35, 37). One study relied on the Likert version of the Western Ontario and McMaster Universities Osteoarthritis Index pain subscale following an individualized, progressive exercise regimen (37). The other study employed the Fugl-Meyer Test after participants underwent mirror therapy (35). Both studies reported no significant difference between individual and group therapy regarding pain. Based on low-level evidence (GRADE) because of a serious risk of bias and small sample sizes in the studies, observed outcomes for pain did not show clear differences between group and individual therapy.

#### 3.4.5 Quality of life and psychosocial factors

Quality of life and psychosocial factors were evaluated in five studies including 391 patients (31, 33–35, 37). Four of these studies evaluated (health-related) quality of life using the Pediatric Quality of Life Inventory (31), the McMaster Toronto Arthritis Questionnaire and EuroQol (37), the King's Health Questionnaire (34), or the Stroke Impact Scale (35). Three studies (31, 35, 37) suggested comparable results in quality of life after intervention between individual and group therapy. However, one study reported differences in favor of individual therapy in personal relationships and emotions domains (34). A study additionally evaluated changes in health beliefs and self-efficacy using the Exercise Health Beliefs and Self-Efficacy Questionnaire and anxiety and depression using the Hospital Anxiety and Depression Scale. No significant differences were measured between individual and group therapy (37). Another study utilized the Turkish Children's Voice Handicap Index−10 to assess self-perception of voice disorders. There were no statistically relevant differences in scores between participants in individual and group therapy. However, according to the speech and language therapists, group therapy was more enjoyable for patients, who were also more competitive and motivated (33). Based on very low-level evidence (GRADE) because of a very serious risk of bias and small sample sizes in the studies, observed outcomes for quality of life and psychosocial factors did not show clear differences between group and individual therapy.

#### 3.4.6 Attendance, compliance, satisfaction, and process indicators

Three studies, including 81 patients, evaluated the difference between individual and group therapies in terms of attendance, compliance, satisfaction, and process indicators (31, 32, 34). Two studies measured satisfaction with treatment using a questionnaire. There was no significant evidence of between-group differences in satisfaction in either study (32, 34). One study recorded home exercise compliance with a logbook. Home exercise compliance suggested comparable results for group and individual therapy (32). In one study, the staff burden of all therapists was evaluated using a self-reported questionnaire and waiting days for patients from the date of recommendation for therapy to the beginning and nonattendance were measured. Group therapy participants had a shorter wait time before starting the program compared to those participating in individual therapy. Thus, earlier intervention was allowed by group therapy, while the staff burden was not increased compared to individual therapy. The mean number of nonattendances appeared to be similar for group and individual therapy (31). Based on very low-level evidence (GRADE) because of a very serious risk of bias and small sample sizes in the studies, observed outcomes for attendance, compliance, satisfaction, and process indicators did not show clear differences between group and individual therapy.

### 3.5 Certainty of evidence

Table 3 provides an overview of the assessment of certainty. As with the results of syntheses, the individual studies were assigned to outcomes and evaluated as a whole domain. Due to the different outcomes and measurement methods, the pooled effect size with its confidence interval could not be analyzed. Consequently, a narrative assessment of the quality of the evidence was conducted. Because of the high overall risk of bias in individual studies, a one-to-two-level decrease was performed in all outcome domains. The results were imprecise if the number of participants was less than 400 (41), which also lowered the quality of the evidence in almost all outcome domains. The results were consistent across the outcome domains, and the evidence directly answered the healthcare question. Therefore, inconsistency and indirectness were rated as not serious. Publication bias was not considered a serious concern. The review addressed a comparison of group and individual therapy rather than a commercially driven intervention, reducing the likelihood of publication being influenced by financial interests. In addition, a comprehensive literature search was conducted across multiple databases using predefined eligibility criteria, and no indications of missing or selectively published evidence were identified. Although publication bias can never be completely excluded, there was insufficient evidence to justify downgrading the certainty of the evidence for this domain.

**Table 3:** GRADE quality assessment for each outcome domain.

Outcome	No. of studies (No. of participants)	Risk of bias	Inconsistency	Indirectness	Imprecision	Dissemination bias	Certainty
Motor function, physical performance and physiological parameters (28–32, 34, 35, 37, 38)	9 (533)	Very serious (- 2): Most studies (6/9) have a high risk of bias	Not serious: (−0)	Not serious: (−0)	Not serious (−0)	Undetected (−0)	⊕⊕○○ Low
Voice-related outcomes (33, 36)	2 (40)	Very serious (−2): The studies have a high risk of bias	Not serious: (−0)	Not serious: (−0)	Serious (−1): Cumulative sample size less than *n* = 400	Undetected (−0)	⊕○○○ Very low
Adaptive behavior and activities of daily living (31, 35, 37)	3 (341)	Very serious (−2): Most studies (2/3) have a high risk of bias	Not serious: (−0)	Not serious: (−0)	Serious (−1): Cumulative sample size less than *n* = 400	Undetected (−0)	⊕○○○ Very low
Pain (35, 37)	2 (317)	Serious (−1): One study has a high risk of bias	Not serious: (−0)	Not serious: (−0)	Serious (−1): Cumulative sample size less than *n* = 400	Undetected (−0)	⊕⊕○○ Low
Quality of life and psychosocial factors (31, 33–35, 37)	5 (391)	Very serious (−2): Most studies (4/5) have a high risk of bias	Not serious: (−0)	Not serious: (−0)	Serious (−1): Cumulative sample size less than *n* = 400	Undetected (−0)	⊕○○○ Very low
Attendance, compliance, satisfaction, and process indicators (31, 32, 34)	3 (81)	Very serious (−2): Most studies (2/3) have a high risk of bias	Not serious: (−0)	Not serious: (−0)	Serious (−1): Cumulative sample size less than *n* = 400	Undetected (−0)	⊕○○○ Very low

The assessment resulted in low or very low overall certainty in all outcome domains. For this reason, the certainty of the evidence regarding the effects of therapeutic services on all outcomes was considered (very) low.

## 4 Discussion

To the best of current knowledge, this is the first systematic review to compare similar interventions between individual and group therapies in the three therapeutic services physiotherapy, occupational therapy and speech therapy. A total of eight studies from the field of physiotherapy, two from the field of speech therapy, and one from the field of occupational therapy were included in this review. Each of the included studies was a randomized controlled trial with identical interventions to determine differences in outcome measures between individual and group therapy. Previous reviews have not considered all three therapeutic service areas and compared identical interventions between individual and group therapies.

Evidence regarding the relative effectiveness of group vs. individual therapy remains limited due to substantial methodological constraints in the included studies. For most outcome domains, reported results did not demonstrate statistically significant differences between participants receiving group and individual therapy post-intervention. Only 4 of the 76 outcome measures showed better results in individual therapy than in group therapy. Conversely, 5 of the 76 outcome measures yielded better results in group therapy than in individual therapy. However, these were only single outcomes out of several per study. The majority (67 of 76) of outcome measures suggested an absence of clear differences when comparing individual and group therapy. When interpreting the results, the lack of significant differences between individual and group therapy should be considered in the context of the small study population. In the studies by Hung et al. (32) or Zamani et al. (36), no significant differences between the groups were found. Upon closer examination of the measurements, individual and group therapy actually appear quite similar, suggesting that the differences are small. Likewise, there are no statistically significant differences in the studies of Hirschmann et al. (31) (except for number of waiting days) or Song et al. (38), even though differences between individual and group therapies were measured. Most studies were only adequately powered to detect medium or large effects, so small, potentially clinically meaningful, effects are likely to remain undetected. Statistical significance depends primarily on sample size and power, whereas clinical significance reflects whether observed differences reach a magnitude that matters for patient care. Therefore, the absence of significant differences does not imply the actual equality of effects (“no difference”), but rather the absence of a detectable effect under the given methodological conditions of the included studies (42). Crucially, this must not be interpreted as evidence of equivalence between group and individual therapy. Observing similar mean outcomes is different from proving equal effectiveness. Rather than establishing therapeutic parity, non-significant findings leave open the possibility that clinically relevant distinctions remain unexamined or that no such differences exist even under improved methodological conditions.

Other reviews that compare the effectiveness of individual and group therapies for therapeutic services come to similar conclusions. In a systematic review examining the effectiveness of individual vs. group therapy in children with developmental coordination disorder, both types of interventions showed overall improvements. However, due to the large heterogeneity and the rather low quality of the studies, the research question of whether individual or group therapies are better could not be answered (43). Hancock et al. compared individual and group therapy to support recovery from stroke and ischemic heart disease. Although showing that there were no consistent differences in the measured health outcomes, the effectiveness of individual and group therapy due to the small sample size in many of the included articles is unclear (44). The systematic review and meta-analysis by McNally et al. compared the delivery of upper limb interventions through individual and group therapy for adults after a stroke. The findings indicate that group therapy might be as effective as individual therapy. At the same time, the authors also point out that the results are limited by the small number of studies included, the small sample size, and the heterogeneity of the studies (45). Similarly, Robertson et al. found that both individual and group therapy showed comparable effectiveness in treating urinary incontinence and low back pain. Nevertheless, Robertson et al. also acknowledge that the results are limited by the quantity and quality of the available evidence (46). Consistent with the findings of this review, the available evidence did not reveal consistent differences in health outcomes between group and individual therapy. Nevertheless, the available evidence remains too limited to draw firm conclusions about comparability. The comparison of therapeutic service delivery formats therefore remains uncertain due to insufficient evidence quality.

The findings of this review are limited in their generalizability due to the significant heterogeneity among the studies concerning diseases, interventions, endpoints, and data collection instruments. The participants of the studies have different diseases and disease progressions, which limits the applicability to other patients, indications and therapies. These variations reflect fundamental differences in therapeutic contexts that constrain the interpretability and transferability of pooled results. Consequently, broad conclusions across diagnostic categories, patient subgroups, and therapeutic disciplines should be drawn with caution. The observed lack of significant differences between group and individual therapy may partly stem from methodological inconsistencies rather than true therapeutic parity. Therefore, generalizability to specific clinical settings or indication profiles remains uncertain.

Due to the high heterogeneity of the studies, the research question of which indications are suitable for group therapy cannot be answered. Consequently, this review does not determine which other diseases within therapeutic services may be suitable for group therapy. Instead, it suggests that group therapies might be suitable for a wide range of different diseases. Further research is needed to identify which other diseases can be treated with group therapy. Unfortunately, no studies on digital therapeutic services could be included. While the PICO framework in this study initially permitted inclusion of both in-person and digital therapy, it is crucial to note that during the screening process, no studies using video therapy (according to the German healthcare system) that met the other inclusion criteria were identified. Consequently, all analyzed data derive exclusively from in-person therapeutic interventions. Due to this limitation, the available evidence does not permit conclusions about the effectiveness of digital group therapies in physiotherapy, occupational therapy and speech therapy.

Of the studies included in this review, only one evaluated process indicators. This study showed that group therapy participants had shorter waiting times, and more patients could be treated at the same time, without increasing staff burden compared to individual therapy (31). Unfortunately, none of the studies that met our inclusion criteria compared the costs of individual and group therapies. The findings of other reviews indicate that group therapy may be more cost-effective than individual therapy (46). Given the limited resources in the healthcare system, group therapies in therapeutic services may be an effective way to treat multiple patients simultaneously with fewer resources. To provide further evidence, future studies should consider the effectiveness of group therapy as well as its cost. These studies should have an adequate sample size to actually determine with high evidence whether there are no differences between individual and group therapies. Particular attention should be directed toward speech therapy and occupational therapy, where current evidence remains scarce.

Several limitations should be considered when interpreting the results. Overall, only eleven studies were included in this review, with pronounced imbalance across therapeutic services. Of these, only two were speech therapy studies and only one was occupational therapy study. Physiotherapy dominated the evidence base. In addition, there were several pilot studies with only a small number of participants. The limited number of studies, particularly in the areas of speech and occupational therapy, as well as the small patient sample sizes, may limit the generalizability of the results to other areas of therapeutic services and indications. Particularly for speech therapy and occupational therapy, conclusions must be interpreted with caution given the limited evidence pool.

Furthermore, only RCTs were included in which the interventions in individual and group therapy were identical. Although this approach helps to exclude confounders and systematic biases, standardized treatment protocols do not align with actual therapeutic services. Notwithstanding, the implementation of group therapies may prove challenging due to differences between healthcare systems among countries.

This review considered various therapeutic services for different diseases and patient populations. Therefore, the studies involved various interventions, encompassing distinct therapeutic techniques, session frequencies, and treatment durations across different settings. Further complicating quantitative synthesis were outcome-related inconsistencies arising from diverse endpoints measured using a variety of instruments. These combined factors rendered quantitative pooling via meta-analysis methodologically inappropriate, as aggregated effect estimates would lack validity and interpretability. Consequently, performing a meta-analysis was not feasible. A comparison of the true effectiveness of individual and group therapy remains to be conducted.

When interpreting the results, it is important to consider the high overall risk of bias (high risk in eight out of eleven studies), as this imposes limitations on the interpretation of the evidence. Due to the high risk of bias in the studies, the certainty of evidence in the outcome domains is also low. A two-level decrease in the certainty of evidence was made in almost all outcome domains based on the high risk of bias. Although the other four criteria led to few downgrades, all outcome domains have low or very low overall evidence certainty. Accordingly, the evidence is (very) uncertain.

This review cannot answer the question of the effectiveness of digital group therapies. Future studies are required to research the effectiveness of digital group therapies compared to digital individual therapies for therapeutic services.

Another limitation concerns the absence of prospective protocol registration. The review protocol was not registered with a public repository prior to study initiation and conduct. Registration would have enhanced methodological transparency.

## 5 Conclusion

This review found no consistent evidence of superior effectiveness for either group or individual therapy in therapeutic services. However, due to the predominance of low-quality studies with limited sample sizes, firm conclusions about comparative performance are precluded. Results should therefore be interpreted with considerable caution. The absence of statistically significant differences cannot serve as validation of comparable effectiveness between group and individual therapy. Due to the small number of studies and the low patient enrollment in each, especially for (digital) speech and occupational therapy, further research in this area is needed. Future studies, utilizing strong methodological frameworks and sufficiently powered samples, should consider costs in addition to treatment effects to determine whether group therapies are resource-efficient treatment options when non-inferiority is demonstrated.

## Data Availability

The original contributions presented in the study are included in the article/Supplementary Material, further inquiries can be directed to the corresponding author.
