# Supplementary material for: Comparison of group and individual therapy in physiotherapy, occupational therapy, and speech therapy: a systematic review
Source: Front Rehabil Sci. 2026 Sep 29;7:1904321. doi: 10.3389/fresc.2026.1904321 (PMC13658143; doi:10.3389/fresc.2026.1904321)
Supplement: Supplementary file 1 [file Supplementaryfile1.docx]

Supplementary Material

Supplementary Table S1. PRISMA 2020 checklist

| **Section and Topic** | **Item #** | **Checklist item** | **Location where item is reported** |
| --- | --- | --- | --- |
| **TITLE** | | |  |
| Title | 1 | Identify the report as a systematic review. | 1 |
| **ABSTRACT** | | |  |
| Abstract | 2 | See the PRISMA 2020 for Abstracts checklist. | 1 |
| **INTRODUCTION** | | |  |
| Rationale | 3 | Describe the rationale for the review in the context of existing knowledge. | 2 |
| Objectives | 4 | Provide an explicit statement of the objective(s) or question(s) the review addresses. | 2 |
| **METHODS** | | |  |
| Eligibility criteria | 5 | Specify the inclusion and exclusion criteria for the review and how studies were grouped for the syntheses. | 3 |
| Information sources | 6 | Specify all databases, registers, websites, organisations, reference lists and other sources searched or consulted to identify studies. Specify the date when each source was last searched or consulted. | 3 |
| Search strategy | 7 | Present the full search strategies for all databases, registers and websites, including any filters and limits used. | 4 |
| Selection process | 8 | Specify the methods used to decide whether a study met the inclusion criteria of the review, including how many reviewers screened each record and each report retrieved, whether they worked independently, and if applicable, details of automation tools used in the process. | 4 |
| Data collection process | 9 | Specify the methods used to collect data from reports, including how many reviewers collected data from each report, whether they worked independently, any processes for obtaining or confirming data from study investigators, and if applicable, details of automation tools used in the process. | 4 |
| Data items | 10a | List and define all outcomes for which data were sought. Specify whether all results that were compatible with each outcome domain in each study were sought (e.g. for all measures, time points, analyses), and if not, the methods used to decide which results to collect. | 4 |
|  | 10b | List and define all other variables for which data were sought (e.g. participant and intervention characteristics, funding sources). Describe any assumptions made about any missing or unclear information. | 4 |
| Study risk of bias assessment | 11 | Specify the methods used to assess risk of bias in the included studies, including details of the tool(s) used, how many reviewers assessed each study and whether they worked independently, and if applicable, details of automation tools used in the process. | 5 |
| Effect measures | 12 | Specify for each outcome the effect measure(s) (e.g. risk ratio, mean difference) used in the synthesis or presentation of results. | 5 |
| Synthesis methods | 13a | Describe the processes used to decide which studies were eligible for each synthesis (e.g. tabulating the study intervention characteristics and comparing against the planned groups for each synthesis (item #5)). | 5 |
|  | 13b | Describe any methods required to prepare the data for presentation or synthesis, such as handling of missing summary statistics, or data conversions. |  |
|  | 13c | Describe any methods used to tabulate or visually display results of individual studies and syntheses. |  |
|  | 13d | Describe any methods used to synthesize results and provide a rationale for the choice(s). If meta-analysis was performed, describe the model(s), method(s) to identify the presence and extent of statistical heterogeneity, and software package(s) used. |  |
|  | 13e | Describe any methods used to explore possible causes of heterogeneity among study results (e.g. subgroup analysis, meta-regression). |  |
|  | 13f | Describe any sensitivity analyses conducted to assess robustness of the synthesized results. |  |
| Reporting bias assessment | 14 | Describe any methods used to assess risk of bias due to missing results in a synthesis (arising from reporting biases). | 5 |
| Certainty assessment | 15 | Describe any methods used to assess certainty (or confidence) in the body of evidence for an outcome. | 5 |
| **RESULTS** | | |  |
| Study selection | 16a | Describe the results of the search and selection process, from the number of records identified in the search to the number of studies included in the review, ideally using a flow diagram. | 5 |
|  | 16b | Cite studies that might appear to meet the inclusion criteria, but which were excluded, and explain why they were excluded. | 6 |
| Study characteristics | 17 | Cite each included study and present its characteristics. | 6 |
| Risk of bias in studies | 18 | Present assessments of risk of bias for each included study. | 7 |
| Results of individual studies | 19 | For all outcomes, present, for each study: (a) summary statistics for each group (where appropriate) and (b) an effect estimate and its precision (e.g. confidence/credible interval), ideally using structured tables or plots. | 7 |
| Results of syntheses | 20a | For each synthesis, briefly summarise the characteristics and risk of bias among contributing studies. | 7 |
|  | 20b | Present results of all statistical syntheses conducted. If meta-analysis was done, present for each the summary estimate and its precision (e.g. confidence/credible interval) and measures of statistical heterogeneity. If comparing groups, describe the direction of the effect. |  |
|  | 20c | Present results of all investigations of possible causes of heterogeneity among study results. |  |
|  | 20d | Present results of all sensitivity analyses conducted to assess the robustness of the synthesized results. |  |
| Reporting biases | 21 | Present assessments of risk of bias due to missing results (arising from reporting biases) for each synthesis assessed. | 7 |
| Certainty of evidence | 22 | Present assessments of certainty (or confidence) in the body of evidence for each outcome assessed. | 1ß |
| **DISCUSSION** | | |  |
| Discussion | 23a | Provide a general interpretation of the results in the context of other evidence. | 10 |
|  | 23b | Discuss any limitations of the evidence included in the review. | 12 |
|  | 23c | Discuss any limitations of the review processes used. | 12 |
|  | 23d | Discuss implications of the results for practice, policy, and future research. | 12 |
| **OTHER INFORMATION** | | |  |
| Registration and protocol | 24a | Provide registration information for the review, including register name and registration number, or state that the review was not registered. |  |
|  | 24b | Indicate where the review protocol can be accessed, or state that a protocol was not prepared. |  |
|  | 24c | Describe and explain any amendments to information provided at registration or in the protocol. |  |
| Support | 25 | Describe sources of financial or non-financial support for the review, and the role of the funders or sponsors in the review. | 13 |
| Competing interests | 26 | Declare any competing interests of review authors. | 13 |
| Availability of data, code and other materials | 27 | Report which of the following are publicly available and where they can be found: template data collection forms; data extracted from included studies; data used for all analyses; analytic code; any other materials used in the review. |  |

*From:*  Page MJ, McKenzie JE, Bossuyt PM, Boutron I, Hoffmann TC, Mulrow CD, et al. The PRISMA 2020 statement: an updated guideline for reporting systematic reviews. BMJ 2021;372:n71. doi: 10.1136/bmj.n71. This work is licensed under CC BY 4.0. To view a copy of this license, visit <https://creativecommons.org/licenses/by/4.0/>

Supplementary Table S2. Search strings and number of hits

| **EMBASE**  **Date of search: May 7, 2025** | | |
| --- | --- | --- |
| **Nr.** | **Terms** | **Hits** |
| 1 | 'group-based':ti,ab | 21.184 |
| 2 | 'group session*':ti,ab | 9.008 |
| 3 | 'group therap*':ti,ab | 8.909 |
| 4 | 'group intervention*':ti,ab | 8.402 |
| 5 | 'group treatment*':ti,ab | 8.203 |
| 6 | 'group setting*':ti,ab | 4.018 |
| 7 | 'group care'/exp | 20 |
| **8** | **1 OR 2 OR 3 OR 4 OR 5 OR 6 OR 7** | **56.611** |
|  | 'group-based':ti,ab OR 'group session*':ti,ab OR 'group therap*':ti,ab OR 'group intervention*':ti,ab OR 'group treatment*':ti,ab OR 'group setting*':ti,ab OR 'group care'/exp |  |
| 9 | 'speech and language rehabilitation'/exp | 25.700 |
| 10 | 'speech therap*':ti,ab | 7.565 |
| 11 | 'language therap*':ti,ab | 4.923 |
| 12 | 'voice therap*':ti,ab | 1.453 |
| 13 | 'logop*':ti,ab | 2.132 |
| 14 | 'swallowing therap*':ti,ab | 498 |
| 15 | 'myofunctional therap*':ti,ab | 463 |
| 16 | 'voice training':ti,ab | 346 |
| 17 | 'speech correction':ti,ab | 82 |
| 18 | 'swallowing therapy'/exp | 37 |
| **19** | **9 OR 10 OR 11 OR 12 OR 13 OR 14 OR 15 OR 16 OR 17 OR 18** | **33.893** |
|  | 'speech and language rehabilitation'/exp OR 'speech therap*':ti,ab OR 'language therap*':ti,ab OR 'voice therap*':ti,ab OR 'logop*':ti,ab OR 'swallowing therap*':ti,ab OR 'myofunctional therap*':ti,ab OR 'voice training':ti,ab OR 'speech correction':ti,ab OR 'swallowing therapy'/exp |  |
| 20 | 'physiotherapy'/exp | 128.596 |
| 21 | 'physiotherap*':ti,ab | 67.406 |
| 22 | 'physical therap*':ti,ab | 49.324 |
| 23 | 'exercise therap*':ti,ab | 7.673 |
| 24 | 'manual therap*':ti,ab | 4.982 |
| 25 | 'physiatric*':ti,ab | 721 |
| **26** | **20 OR 21 OR 22 OR 23 OR 24 OR 25** | **185.220** |
|  | 'physiotherapy'/exp OR 'physiotherap*':ti,ab OR 'physical therap*':ti,ab OR 'exercise therap*':ti,ab OR 'manual therap*':ti,ab OR 'physiatric*':ti,ab |  |
| 27 | 'daily life activity'/exp | 130.318 |
| 28 | 'activit* of daily living':ti,ab | 56.426 |
| 29 | 'occupational therapy'/exp | 31.712 |
| 30 | 'occupational therap*':ti,ab | 28.715 |
| 31 | 'ADL':ti,ab | 24.019 |
| 32 | 'ergotherap*':ti,ab | 708 |
| **33** | **27 OR 28 OR 29 OR 30 OR 31 OR 32** | **187.859** |
|  | 'daily life activity'/exp OR 'activit* of daily living':ti,ab OR 'occupational therapy'/exp OR 'occupational therap*':ti,ab OR 'ADL':ti,ab OR 'ergotherap*':ti,ab |  |
| **34** | **19 OR 26 OR 33** | **379.796** |
| **35** | **8 AND 34** | **3.197** |
| Search string EMBASE:  ('group-based':ti,ab OR 'group session*':ti,ab OR 'group therap*':ti,ab OR 'group intervention*':ti,ab OR 'group treatment*':ti,ab OR 'group setting*':ti,ab OR 'group care'/exp) AND ('speech and language rehabilitation'/exp OR 'speech therap*':ti,ab OR 'language therap*':ti,ab OR 'voice therap*':ti,ab OR 'logop*':ti,ab OR 'swallowing therap*':ti,ab OR 'myofunctional therap*':ti,ab OR 'voice training':ti,ab OR 'speech correction':ti,ab OR 'swallowing therapy'/exp OR 'physiotherapy'/exp OR 'physiotherap*':ti,ab OR 'physical therap*':ti,ab OR 'exercise therap*':ti,ab OR 'manual therap*':ti,ab OR 'physiatric*':ti,ab OR 'daily life activity'/exp OR 'activit* of daily living':ti,ab OR 'occupational therapy'/exp OR 'occupational therap*':ti,ab OR 'ADL':ti,ab OR 'ergotherap*':ti,ab) | | |
| **PubMed**  **Date of search: May 7, 2025** | | |
| **Nr.** | **Terms** | **Hits** |
| 1 | "group-based"[Title/Abstract] | 14.063 |
| 2 | "group therap*"[Title/Abstract] | 5.903 |
| 3 | "group session*"[Title/Abstract] | 5.836 |
| 4 | "group intervention*"[Title/Abstract] | 4.767 |
| 5 | "group treatment*"[Title/Abstract] | 3.505 |
| 6 | "group setting*"[Title/Abstract] | 2.006 |
| **7** | **1 OR 2 OR 3 OR 4 OR 5 OR 6** | **33.840** |
|  | "group-based" [Title/Abstract] OR "group therap*"[Title/Abstract] OR "group session*" [Title/Abstract] OR "group intervention*" [Title/Abstract] OR "group treatment*" [Title/Abstract] OR "group setting*" [Title/Abstract] |  |
| 8 | "speech therapy"[MeSH] | 7.182 |
| 9 | "speech therap*"[Title/Abstract] | 4.904 |
| 10 | "language therap*"[Title/Abstract] | 3.111 |
| 11 | "voice therap*"[Title/Abstract] | 1.339 |
| 12 | "logop*"[Title/Abstract] | 1.252 |
| 13 | "voice training"[Title/Abstract] | 449 |
| 14 | "myofunctional therap*"[Title/Abstract] | 414 |
| 15 | "swallowing therap*"[Title/Abstract] | 294 |
| 16 | "speech correction"[Title/Abstract] | 72 |
| **17** | **8 OR 9 OR 10 OR 11 OR 12 OR 13 OR 14 OR 15 OR 16** | **15.716** |
|  | "speech therapy"[MeSH] OR "speech therap*"[Title/Abstract] OR "language therap*"[Title/Abstract] OR "voice therap*"[Title/Abstract] OR "logop*"[Title/Abstract] OR "voice training"[Title/Abstract] OR "myofunctional therap*"[Title/Abstract] OR "swallowing therap*"[Title/Abstract] OR "speech correction"[Title/Abstract] |  |
| 18 | "physical therapy modalities"[MeSH] | 192.650 |
| 19 | "physiotherap*"[Title/Abstract] | 40.326 |
| 20 | "physical therap*"[Title/Abstract] | 37.937 |
| 21 | "exercise therap*"[Title/Abstract] | 7.801 |
| 22 | "manual therap*"[Title/Abstract] | 4.233 |
| 23 | "physical therapy specialty"[MeSH] | 3.172 |
| 24 | "physiatric*"[Title/Abstract] | 471 |
| **25** | **18 OR 19 OR 20 OR 21 OR 22 OR 23 OR 24** | **243.164** |
|  | "physical therapy modalities"[MeSH] OR "physiotherap*"[Title/Abstract] OR "physical therap*"[Title/Abstract] OR "exercise therap*"[Title/Abstract] OR "manual therap*"[Title/Abstract] OR "physical therapy specialty"[MeSH] OR "physiatric*"[Title/Abstract] |  |
| 26 | "activit* of daily living"[Title/Abstract] | 42.428 |
| 27 | "occupational therap*"[Title/Abstract] | 20.672 |
| 28 | "occupational therapy"[MeSH] | 15.872 |
| 29 | "ADL"[Title/Abstract] | 15.396 |
| 30 | "ergotherap*"[Title/Abstract] | 458 |
| **31** | **26 OR 27 OR 28 OR 29 OR 30** | **71.441** |
|  | "activit* of daily living"[Title/Abstract] OR "occupational therap*"[Title/Abstract] OR "occupational therapy"[MeSH] OR "ADL"[Title/Abstract] OR "ergotherap*"[Title/Abstract] |  |
| **32** | **17 OR 25 OR 31** | **317.853** |
|  | "speech therapy"[MeSH] OR "speech therap*"[Title/Abstract] OR "language therap*"[Title/Abstract] OR "voice therap*"[Title/Abstract] OR "logop*"[Title/Abstract] OR "voice training"[Title/Abstract] OR "myofunctional therap*"[Title/Abstract] OR "swallowing therap*"[Title/Abstract] OR "speech correction"[Title/Abstract] OR "physical therapy modalities"[MeSH] OR "physiotherap*"[Title/Abstract] OR "physical therap*"[Title/Abstract] OR "exercise therap*"[Title/Abstract] OR "manual therap*"[Title/Abstract] OR "physical therapy specialty"[MeSH] OR "physiatric*"[Title/Abstract] OR "activit* of daily living"[Title/Abstract] OR "occupational therap*"[Title/Abstract] OR "occupational therapy"[MeSH] OR "ADL"[Title/Abstract] OR "ergotherap*"[Title/Abstract] |  |
| **33** | **7 AND 32** | **2.286** |
| Search string PubMed:  ("group-based"[Title/Abstract] OR "group therap*"[Title/Abstract] OR "group session*"[Title/Abstract] OR "group intervention*"[Title/Abstract] OR "group treatment*"[Title/Abstract] OR "group setting*"[Title/Abstract]) AND ("speech therapy"[MeSH] OR "speech therap*"[Title/Abstract] OR "language therap*"[Title/Abstract] OR "voice therap*"[Title/Abstract] OR "logop*"[Title/Abstract] OR "voice training"[Title/Abstract] OR "myofunctional therap*"[Title/Abstract] OR "swallowing therap*"[Title/Abstract] OR "speech correction"[Title/Abstract] OR "physical therapy modalities"[MeSH] OR "physiotherap*"[Title/Abstract] OR "physical therap*"[Title/Abstract] OR "exercise therap*"[Title/Abstract] OR "manual therap*"[Title/Abstract] OR "physical therapy specialty"[MeSH] OR "physiatric*"[Title/Abstract] OR "activit* of daily living"[Title/Abstract] OR "occupational therap*"[Title/Abstract] OR "occupational therapy"[MeSH] OR "ADL"[Title/Abstract] OR "ergotherap*"[Title/Abstract]) | | |
| **Web of Science**  **Date of search: May 7, 2025** | | |
| **Nr.** | **Terms** | **Hits** |
| 1 | TS=("group-based") | 23.449 |
| 2 | TS=("group therap*") | 9.607 |
| 3 | TS=("group session*") | 6.528 |
| 4 | TS=("group intervention*") | 7.259 |
| 5 | TS=("group treatment*") | 6.612 |
| 6 | TS=("group setting*") | 3.297 |
| **7** | **1 OR 2 OR 3 OR 4 OR 5 OR 6** | **53.659** |
|  | TS=("group-based" OR "group therap*" OR "group session*" OR "group intervention*" OR "group treatment*" OR "group setting*") |  |
| 8 | TS=("speech therap*") | 5.266 |
| 9 | TS=("language therap*") | 3.885 |
| 10 | TS=("voice therap*") | 1.418 |
| 11 | TS=("logop*") | 1.512 |
| 12 | TS=("voice training") | 497 |
| 13 | TS=("myofunctional therap*") | 324 |
| 14 | TS=("swallowing therap*") | 319 |
| 15 | TS=("speech correction") | 119 |
| **16** | **8 OR 9 OR 10 OR 11 OR 12 OR 13 OR 14 OR 15** | **12.565** |
|  | TS=("speech therap*" OR "language therap*" OR "voice therap*" OR "logop*" OR "voice training" OR "myofunctional therap*" OR "swallowing therap*" OR "speech correction") |  |
| 17 | TS=("physiotherap*") | 44.860 |
| 18 | TS=("physical therap*") | 38.438 |
| 19 | TS=("exercise therap*") | 8.005 |
| 20 | TS=("manual therap*") | 5.042 |
| 21 | TS=("physiatric*") | 448 |
| **22** | **17 OR 18 OR 19 OR 20 OR 21** | **85.705** |
|  | TS=("physiotherap*" OR "physical therap*" OR "exercise therap*" OR "manual therap*" OR "physiatric*") |  |
| 23 | TS=("activit* of daily living") | 43.707 |
| 24 | TS=("occupational therap*") | 23.173 |
| 25 | TS=("ADL") | 18.150 |
| 26 | TS=("ergotherap*") | 251 |
| **27** | **23 OR 24 OR 25 OR 26** | **71.888** |
|  | TS=("activit* of daily living" OR "occupational therap*" OR "ADL" OR "ergotherap*") |  |
| **28** | **16 OR 22 OR 27** | **162.270** |
|  | TS=("speech therap*" OR "language therap*" OR "voice therap*" OR "logop*" OR "voice training" OR "myofunctional therap*" OR "swallowing therap*" OR "speech correction" OR "physiotherap*" OR "physical therap*" OR "exercise therap*" OR "manual therap*" OR "physiatric*" OR "activit* of daily living" OR "occupational therap*" OR "ADL" OR "ergotherap*") |  |
| **29** | **7 AND 28** | **1.522** |
| Search string Web of Science  TS=("group-based" OR "group therap*" OR "group session*" OR "group intervention*" OR "group treatment*" OR "group setting*") AND TS=("speech therap*" OR "language therap*" OR "voice therap*" OR "logop*" OR "voice training" OR "myofunctional therap*" OR "swallowing therap*" OR "speech correction" OR "physiotherap*" OR "physical therap*" OR "exercise therap*" OR "manual therap*" OR "physiatric*" OR "activit* of daily living" OR "occupational therap*" OR "ADL" OR "ergotherap*") | | |

Supplementary Table S3. Extraction table

|  | **Number** | **28** | **29** | **30** | **31** | **32** | **37** | **33** | **34** | **38** | **35** | **36** |
| --- | --- | --- | --- | --- | --- | --- | --- | --- | --- | --- | --- | --- |
|  | **Author** | Ciortea et al. | Dorrell et al. | Doussoulin et al. | Hirschmann et al. | Hung et al. | Hurley et al. | Karalı et al. | Pereira et al. | Song et al. | Thieme et al. | Zamani et al. |
|  | **Year** | 2020 | 2020 | 2018 | 2023 | 2010 | 2007 | 2022 | 2011 | 2015 | 2012 | 2018 |
| **Study data** | **Title** | Effects of group physical therapy on the walking speed in patients with Parkinson’s disease | Comparison of individual and group-based load-velocity profiling as a means to dictate training load over a 6-week strength and power intervention | Effects of modified constraint-induced movement therapy in the recovery of upper extremity function affected by a stroke: a single-blind randomized parallel trial-comparing group versus individual intervention | Group versus individual occupational therapy for toddlers with autism as a means to improve access to public healthcare services. Randomised controlled pilot study | EFFECTS OF GROUP-BASED VERSUS INDIVIDUAL-BASED EXERCISE TRAINING ON MOTOR PERFORMANCE IN CHILDREN WITH DEVELOPMENTAL COORDINATION DISORDER: A RANDOMIZED CONTROLLED PILOT STUDY | Clinical effectiveness of a rehabilitation program integrating exercise, self-management, and active coping strategies for chronic knee pain: a cluster randomized trial | Voice Therapy Efficacy in Pediatric Professional Voice Users With Vocal Fold Nodules: A Preliminary Study | Individual and group pelvic floor muscle training versus no treatment in female stress urinary incontinence: a randomized controlled pilot study | Effect of the class and individual applications of task-oriented circuit training on gait ability in patients with chronic stroke | Mirror therapy for patients with severe arm paresis after stroke – a randomized controlled trial | The effects of narrative-based language intervention (NBLI) on spoken narrative structures in Persian-speaking cochlear implanted children: A prospective randomized control trial |
|  | **Country** | Romania | United Kingdom | Chile | Israel | China | United Kingdom | Turkey | Brazil | Korea | Germany | Iran |
| **Study type and design** | **Randomization** | Yes | Yes | Yes | Yes | Yes | Yes | Yes | Yes | Yes | Yes | Yes |
|  | **Intervention group (group therapy) and control group (individual therapy)** | Yes | Yes | Yes | Yes | Yes | Yes | Yes | Yes | Yes | Yes | Yes |
|  | **Data collection period** | November 2014 - July 2017 | NR | NR | September 2018 - January 2020 | May 2007 - December 2007 | August 2000 - October 2004 | NR | August 2008 - December 2009 | NR | NR | NR |
|  | **Setting** | Clinical Rehabilitation Hospital Cluj-Napoca, Cluj county, Romania | NR | Temuco (Chile) Community Rehabilitation Centre | Institute for Child Development associated with Shamir Medical Center, Israel | Pediatric Physiotherapy Outpatient Department of Kowloon Hospital | Rehabilitation Research Unit, Dulwich Community Hospital | Asım Sabri Efendi Quran School | Laboratory for Assessment and Intervention on Women’s Health, Federal University of Sao Carlos | C and S hospitals | Inpatient rehabilitation centre | Three cochlear implantation and auditory-verbal rehabilitation centers (Amir-Alam Hospital, Pedjvak Center, Loghman Hospital) in the city of Tehran, Iran |
| **Study population** | **Number of patients group therapy** | 30 | NR | 24 | 14 | 12 | 132 | 8 | 17 | 10 | 21 | 12 |
|  | **Drop-out group therapy** | NR | NR | NR | 4 | 0 | 24 | 2 | 2 | NR | 5 | NR |
|  | **Number of patients individual therapy** | 30 | NR | 12 | 10 | 11 | 146 | 8 | 17 | 10 | 18 | 12 |
|  | **Drop-out individual therapy** | NR | NR | NR | 0 | 0 | 25 | 1 | 2 | NR | 3 | NR |
|  | **Inclusion criteria** | (1) stable medication usage; (2) Hoehn and Yahr stage 2, 3 or 4; (3) ability to walk independently or by using an assistive walking device; (4) age 50 to 70 years; subjects diagnosed with typical, idiopathic parkinson disease. | All participants had at least 2 years of resistance training experience and had been engaged in continuous resistance training for at least 6 months prior to the programme start date. | (1) Age range between 30 and 80 years; (2) Presenting a single stroke confirmed by computed tomography, with a greater evolution period than 6 months; (3) Obtaining between five and 14 points on the National Institutes of Health Stroke Scale, less than two points on the Modified Ashworth Scale, and less than four points on the visual analog scale; (4) Being able to perform a functional test of 20° wrist extension and 10° finger extension. | Toddlers (2-4 years) suspected of having ASD as evaluated by a paediatric neurologist. Only toddlers who received a formal ASD diagnosis based on DSM-5 criteria from both a paediatric neurologist and later by a certified psychologist were included. Psychological evaluation was based on DSM-5 criteria, Mullen Scales of Early Learning and Childhood Autism Rating Scale (CARS) questionnaire, second edition. | A child had to be aged between 6 and 10 years with a diagnosis of DCD. The diagnosis was determined following an assessment performed by an interdisciplinary team (comprising a paediatrician, a paediatric neurologist, a physiotherapist, an occupational therapist, and a clinical psychologist) at the Child Assessment Centre according to the criteria stated in the Diagnostic and Statistical Manual of Mental Disorders (DSM-IV-TR) (1). These are: (i) motor coordination substantially below that expected for their age, with a gross motor composite score of < 42 as measured by the Bruininks- Oseretsky Test of Motor Proficiency (13); (ii) motor difficulties that significantly interfere with academic performance or activities of daily living; (iii) motor difficulties that cannot be explained by any medical or neurological disorders (e.g. cerebral palsy); and (iv) an intelligence level within the normal range. | Individuals age 50 years or older who had consulted a primary care physician for mild, moderate, or severe knee pain of 6 months’ duration. Many participants’ condition had been labeled OA based on their clinical presentation and history without attempting to identify the cause of pain using investigations not routinely available to primary care physicians (e.g., radiographs). | Pediatric professional voice users with vocal fold nodules. | Women aged over 18 years, who complained of urinary leakage on stress and who had not undergone physical therapy for UI before. | Patients with hemiplegia who were diagnosed with stroke. | (a) they had to have had a first supratentorial stroke within the previous three months, ensured through the diagnosis of the primary care hospital, (b) be aged between 18 and 80 years and (c) be clinically diagnosed with a severe distal hemiparesis of the arm (Medical Research Council13 grading of 0 or 1 for wrist and finger extensors). | The children who were eligible for the study were pre-lingually severely to profoundly deaf and had received unilateral 22-channel Nucleus cochlear implants before the age of 4 years. They and their families spoke Persian as their preferred language. All CI children had attended speech therapy sessions for at least 12 months before they participated in the study. Thus, they were able to produce simple sentences with speech intelligibility of more than 85% according to the Persian version of the speech intelligibility measurement test as developed by Heydari et al. None of them were able to narrate a story or produce spoken narrative. All participants had a normal range of IQ. None of the children had noticeable accompanying developmental disabilities or co-morbidities such as attention deficit and hyperactivity disorder or autism spectrum disorder. |
|  | **Exclusion criteria** | (5) severe cognitive impairments (Mini-Mental State Examination - MMSE score, ≥24); (6) other severe neurologic, cardiopulmonary or orthopedic disorders; (7) having participated in a PT or rehabilitation program in the previous 2 months. | NR | Patients with sensory compromises (visual-auditory) and orthopedic limitations (use of walking stick) were excluded. | Toddlers were excluded from the study if their later psychological evaluation did not suggest a diagnosis of ASD, in case they had visual and/or hearing impairments, or if they were admitted to a special education kindergarten during the study period (occupational therapy services are provided in the educational system). | (i) had received or were undergoing physical therapy or occupational therapy; (ii) had any profound visual or hearing deficiencies that could not be corrected by external devices; and (iii) demonstrated excessive disruptive behaviour. Each child referred to the Kowloon Hospital by the Child Assessment Centre was further screened by our research personnel using the Motor Assessment Battery for Children (MABC) (14). Children with an MABC total impairment score of greater than the 15th percentile (i.e. those that fell into the no motor difficulty category) were excluded. | Lower limb arthroplasty, physiotherapy for knee pain in the preceding 12 months, intraarticular injections in the preceding 6 months, unstable medical conditions, inability/unwillingness to exercise, wheelchair dependence, and inability to understand English. Participants were not excluded if they used assistive walking devices; had stable comorbidities common in this age group (e.g., type II diabetes, cardiovascular or respiratory disorders); or had back, lower, or upper limb pain. | NR | Women with symptoms of urgency urinary incontinence and mixed urinary incontinence were excluded. Exclusion criteria also included latex allergies, vaginal or urinary infections, pelvic organ prolapse greater than grade II on Baden– Walker classification system, cognitive or neurological disorder, uncontrolled hypertension and inability to carry out the evaluation or treatment. | Ten patients were excluded from the pool of subjects due to health problems. | (a) visual impairments which may limit the participation in mirror therapy, (b) severe cognitive and/or language deficits that could prevent them from following instructions, (c) other neurological or musculoskeletal impairments of the upper extremity unrelated to stroke and (d) a severe visuospatial neglect (clinically evaluation: the patients did not turn their face to an examiner on their contralesional side if they were asked to do so). | The exclusion criteria were inability of a child to follow instructions or inability of the child or family to participate continuously, failure to attend the intervention sessions for two weeks or more because of prosthesis trouble or sickness (except common colds). |
| **Intervention and control** | **Therapeutic service** | Physiotherapy | Physiotherapy | Physiotherapy | Occupational therapy | Physiotherapy | Physiotherapy | Speech therapy | Physiotherapy | Physiotherapy | Physiotherapy | Speech therapy |
|  | **Duration** | 10 sessions of physical therapy, each 1.5 hour long, on a daily basis, for 2 weeks | Two training sessions each week, over a 6-week mesocycle | The protocol was administered six times | 12 weekly sessions intervention period over 3 months | Weekly session for 8 consecutive weeks | 12 supervised sessions twice weekly for 6 weeks | 6 weeks | 12 sessions, with two 1 h sessions per week and total of 6 weeks of treatment | 30 minutes a day, three times a week for four weeks | 30 minutes daily with a total of 20 sessions during five weeks | 24 1-h sessions over the course of 8 weeks total of 1440 min |
|  | **Description of group therapy** | All treatment sessions were conducted at the same time of the day throughout the study, in the morning, 60-90 minutes after intake of pharmaceutical treatment for PD. For the GPT, there were groups of 6 patients, supervised by 1 physical therapist. The group sizes were kept small to promote efficiency and motivation. The patients undertaking IPT were alone with the physical therapist during the sessions. Both groups were treated in the same physical therapy room setting. The rehabilitation protocol for IPT consisted of cardiovascular warm-up activities, stretching exercises, strengthening exercises, functional, gait and balance training, recreational games, and ended with relaxation exercises. In addition, the GPT protocol followed the exact same pattern, except for 5-10 minute breaks for informal socialization between participants, at the beginning of the session, mid-session and at the end of the session. | Individual load-velocity (ILVP) and group load-velocity profiling (GLVP) intervention, over a 6-week lower body strength and power phase.Prior to all testing and training sessions participants were supervised during a standardised warm-up, consisting of five minutes of stationary cycling (Wattbike; UK; 60 rpm, 60 W), followed by an additional five minutes of self-prescribed dynamic mobility work. All participants completed two training sessions each week, over a 6-week mesocycle focusing on lower-body strength and power, before repeating the testing battery post-intervention. For both training groups, the base programme followed a wave-like periodisation structure. In addition to the back squat, supplementary exercises were included in the training intervention. To ensure consistency between the two groups, sets and repetitions were equated, with load dictated via specific equations, using body mass, or through use of repetitions in reserve approach. For all movements, a RIR of 2–3 repetitions was detailed to the participants (excluding back squat and box jump). | On each occasion, the four patients included in the group therapy worked together in the same place. The intervention protocol was applied in both groups for 3 h daily for 10 days by an expert physical therapist and student assistants.The protocols applied included three main elements in both modalities: (a) Repetitive training oriented at the task: during each session, patients performed repetitive exercises that simulated ADL, oriented at training the affected upper extremity. Exercises were performed using shaping, a behavioral technique aimed at increasing the amount and extent of limb use by performing motor tasks. To do this, 30 tasks were performed involving the shoulder joints, elbows, wrists, and hands. Each functional activity is practiced in 10 sets of 10 attempts for 60–120 s. (b) Induction to use of the affected upper extremity: motor restriction of the unharmed upper extremity was performed using a glove, which eliminates the possibility of using the hand for most of the functional activities, especially when the physical therapist is not present. The goal was to use the glove 70% of daylight hours, avoiding use in activities such as bathing and any activity that posed a danger for the patient. (c) Package transfer: one of the intervention goals is to transfer patient achievements in therapy to the real world. To achieve this objective, a set of behavioral techniques called ‘transfer package’ was used whose objective is to ensure that patients are responsible for their adherence to therapy requirements, ensuring that the patients participate actively in the intervention, without the constant supervision of the physical therapist, a relevant factor in everyday life in which this professional might not be present. The interventions procedure included common activities for both modalities and some exclusive ones, as described below: After patients arrived at the therapy location, the first activity was the Home Diary review. This document describes the tasks (with and without the glove) performed by patients at home during the time while not in therapy, which forms part of the transfer package. In the individual mode, the home diary was reviewed only with the therapist. Subsequently, patients in both modalities performed specific motor tasks. Six previously defined activities were performed that simulated ADL, involving movements of the shoulders, elbows, wrists, and fingers. Each activity has its own requirements, parameters, progressions, and objectives. To avoid fatigue, increased tone, and demotivation, rest periods and stretches were included between each exercise. In the group therapy, free interaction among patients during exercises and breaks was allowed. The motor tasks used the highest percentage of hours during therapy: ~ 70%. Sessions were concluded by defining five specific tasks to perform at home, using the glove, with the aim of promoting the use of the weak arm in off-site therapy. | 12 weekly sessions of group or individual occupational therapy based on the same mode of intervention: Developmental, Individual-Differences and Relationship-based. In both interventions, each session lasted 45 min. Group occupational therapy comprised a series of 12 weekly sessions (intervention period) administered by two occupational therapists over 3 months. Up to four children were included in each group. In the current study, the content and structure of both individual and group interventions were based on the DIR floor-time model, focussing on the first three DIR stages of development: (1) sensory regulation, (2) intimacy and making contact and (3) twoway communication. The principles of the DIR-floor-time therapy session included tuning into the child’s interest in activities while encouraging exploration and problem-solving, following cues of the child while giving meaning to each of the child’s actions (planned, spontaneous or no action) and joining a mutual activity in a playfulness way, including preservative games, encouraging regulation and communication by using facial expression, tone of voice and affect or imitating the child’s actions. In occupational therapy, we use the DIR floortimemethodology to promote the child’s participation in daily activities, play and motor skills while addressing and responding to sensory regulation and communication difficulties. This ‘face-to-face’ mode of intervention consists of pre-planned, semi-spontaneous play sessions in which relationships are built, and functional capacities such as self-regulation, two-way communication, social and emotional engagement, complex thinking and problem-solving are developed. On a table at the side of the room, we placed three transparent cylindrical containers with various threading elements inside and near them a basket with stickers. A barrel and a rocking bowl were placed on the floor in the middle of the room. On the other side of the room, we placed two storage boxes on a mattress, containing cars, dolls and utensils. The occupational therapists were instructed to sit near each station and avoid moving around in order to prevent emotional ‘flooding’ and to support the participants’ regulation. The children were allowed to approach the play stations according to their own preference. To help the children to participate in play, the occupational therapist demonstrated games, for example, tossing balls into the barrel or threading beads from the containers, encouraging the children to join, yet allowing them to choose an activity of their own choice. Because there were up to four children in a group, each occupational therapist played with one or two children at a time, whereas some children played on their own for up to 5 min, and then a therapist approached them. In certain instances, when the therapist noticed that a child was not interested in the stations, additional items were introduced including blocks, cons or scotch balls, depending on the cooperation and engagement in mutual activity. | The subjects underwent a weekly 45-min session of motor skill training for 8 consecutive weeks. The activities involved in the individual training and group training were essentially the same. A variety of functional tasks and exercises was designed to address common motor difficulties faced by children with DCD, such as agility, balance, core stability, and movement coordination. The same exercises were applied to both groups in each treatment session. As the training progressed, the motor tasks were adapted to ensure successful execution of the tasks while providing an adequate challenge to the child’s motor abilities. To maintain the consistency of the treatment approach, both the group-based and individual-based training were conducted by the same physiotherapist, who had many years of experience in paediatric rehabilitation. Each subject was given home exercises to reinforce what had been learned at each session and to increase the exercise frequency. The home exercises were selected from among those used in face-to-face training sessions, and were intended to address the specific areas of motor difficulty experienced by each child. The children were instructed to perform these exercises on a daily basis within the study period. The parents were provided with clear written instructions, and were asked to coach or assist their children in performing the home exercises. The exercise programme was designed to take approximately 20 min to complete. | The content and format were identical for both Indiv-rehab and Grp-rehab, involving 12 supervised sessions (twice weekly for 6 weeks) that combined discussion on specific topics regarding self-management and coping, etc., with an individualized, progressive exercise regimen. To ensure consistency in content and delivery, the same experienced physiotherapist devised, supervised, and progressed all sessions for all participants (NW). | A standardized voice therapy protocol was followed. Each therapy session lasted 30 minutes. Each therapist was present during the therapy sessions.The voice therapy sessions were designed to run over 6 weeks, either individually or as a group. Participants were randomly divided into two groups: receiving either individual voice therapy (eight participants) or group voice therapy (eight participants). | The treatment protocol was carried out as outpatient activities under the supervision of the same physical therapist that carried out the evaluation. The treatment for the GT and IT groups consisted of 12 sessions, with two 1 h sessions per week and total of 6 weeks of treatment. Both groups were taught to contract the pelvic floor muscles correctly, and the proper contractions were confirmed by vaginal palpation. The subjects of GT group were divided into groups of 8–10 people for treatment. During the sessions, volunteers received instructions about anatomy of the pelvic floor muscles and continence mechanisms and carried out exercises to strengthen the pelvic floor muscles in supine, sitting and standing positions. The difficulty degree progressed according to the positions adopted, increasing the number of repetitions and time of sustained contraction. An average of 100 contractions were performed per session, with phasic contractions, lasting three seconds with six seconds of rest, and tonic contractions, lasting 5–10 s followed by 10–20 s of rest [18]. To minimize the muscle fatigue, the resting time was rigidly observed in all sessions and the time of sustained contraction was slowly increased. At the beginning of the individual treatment, the time of sustained contraction was the same recorded for each woman during the initial evaluation. For the GT group, the mean time of the group was considered as the time of sustained contraction. For both groups, the time of sustained contraction was increased by 1 s per week up to 10 s. | Class-based taskoriented circuit training (CTCT) (group III). The TOCT proposed by Salbach et al.4) was modified into a circular mode. The training tasks were sitting in chair and walking, walking over obstacles, carrying goods, turning the goods upside down, and walking fast in a circle. Group III was divided into two groups, each consisting of four to six persons under the supervision of two physiotherapists with more than 10 years of physiotherapy experience each. | Mirror therapy group: Patients in this group underwent mirror therapy within a group therapy protocol (i.e. one therapist treated between 2 and 6 patients at the same time). Mirror therapy in this group followed the same protocol as described for individual therapy. | The instructions and session intensity of the NBLI were similar for both groups, except for their presentation. Both groups also received CST sessions by their clinical practitioners concurrent to the intervention. |
|  | **Group size** | 6 | NR | 4 | Mean of 2,2, up to 4 children in a group | 4-6 | 8 | 4 | 8-10 | Four to six persons | 2-6 | NR |
|  | **Description of indvidual therapy** | All treatment sessions were conducted at the same time of the day throughout the study, in the morning, 60-90 minutes after intake of pharmaceutical treatment for PD. For the GPT, there were groups of 6 patients, supervised by 1 physical therapist. The group sizes were kept small to promote efficiency and motivation. The patients undertaking IPT were alone with the physical therapist during the sessions. Both groups were treated in the same physical therapy room setting. The rehabilitation protocol for IPT consisted of cardiovascular warm-up activities, stretching exercises, strengthening exercises, functional, gait and balance training, recreational games, and ended with relaxation exercises. In addition, the GPT protocol followed the exact same pattern, except for 5-10 minute breaks for informal socialization between participants, at the beginning of the session, mid-session and at the end of the session. | Individual load-velocity (ILVP) and group load-velocity profiling (GLVP) intervention, over a 6-week lower body strength and power phase.Prior to all testing and training sessions participants were supervised during a standardised warm-up, consisting of five minutes of stationary cycling (Wattbike; UK; 60 rpm, 60 W), followed by an additional five minutes of self-prescribed dynamic mobility work. All participants completed two training sessions each week, over a 6-week mesocycle focusing on lower-body strength and power, before repeating the testing battery post-intervention. For both training groups, the base programme followed a wave-like periodisation structure. In addition to the back squat, supplementary exercises were included in the training intervention. To ensure consistency between the two groups, sets and repetitions were equated, with load dictated via specific equations, using body mass, or through use of repetitions in reserve approach. For all movements, a RIR of 2–3 repetitions was detailed to the participants (excluding back squat and box jump). | On each occasion, the four patients included in the group therapy worked together in the same place. The intervention protocol was applied in both groups for 3 h daily for 10 days by an expert physical therapist and student assistants.The protocols applied included three main elements in both modalities: (a) Repetitive training oriented at the task: during each session, patients performed repetitive exercises that simulated ADL, oriented at training the affected upper extremity. Exercises were performed using shaping, a behavioral technique aimed at increasing the amount and extent of limb use by performing motor tasks. To do this, 30 tasks were performed involving the shoulder joints, elbows, wrists, and hands. Each functional activity is practiced in 10 sets of 10 attempts for 60–120 s. (b) Induction to use of the affected upper extremity: motor restriction of the unharmed upper extremity was performed using a glove, which eliminates the possibility of using the hand for most of the functional activities, especially when the physical therapist is not present. The goal was to use the glove 70% of daylight hours, avoiding use in activities such as bathing and any activity that posed a danger for the patient. (c) Package transfer: one of the intervention goals is to transfer patient achievements in therapy to the real world. To achieve this objective, a set of behavioral techniques called ‘transfer package’ was used whose objective is to ensure that patients are responsible for their adherence to therapy requirements, ensuring that the patients participate actively in the intervention, without the constant supervision of the physical therapist, a relevant factor in everyday life in which this professional might not be present. The interventions procedure included common activities for both modalities and some exclusive ones, as described below: After patients arrived at the therapy location, the first activity was the Home Diary review. This document describes the tasks (with and without the glove) performed by patients at home during the time while not in therapy, which forms part of the transfer package. In the individual mode, the home diary was reviewed only with the therapist. Subsequently, patients in both modalities performed specific motor tasks. Six previously defined activities were performed that simulated ADL, involving movements of the shoulders, elbows, wrists, and fingers. Each activity has its own requirements, parameters, progressions, and objectives. To avoid fatigue, increased tone, and demotivation, rest periods and stretches were included between each exercise. In the group therapy, free interaction among patients during exercises and breaks was allowed. The motor tasks used the highest percentage of hours during therapy: ~ 70%. Sessions were concluded by defining five specific tasks to perform at home, using the glove, with the aim of promoting the use of the weak arm in off-site therapy. | 12 weekly sessions of group or individual occupational therapy based on the same mode of intervention: Developmental, Individual-Differences and Relationship-based. In both interventions, each session lasted 45 min. Group occupational therapy comprised a series of 12 weekly sessions (intervention period) administered by two occupational therapists over 3 months. Up to four children were included in each group. In the current study, the content and structure of both individual and group interventions were based on the DIR floor-time model, focussing on the first three DIR stages of development: (1) sensory regulation, (2) intimacy and making contact and (3) twoway communication. The principles of the DIR-floor-time therapy session included tuning into the child’s interest in activities while encouraging exploration and problem-solving, following cues of the child while giving meaning to each of the child’s actions (planned, spontaneous or no action) and joining a mutual activity in a playfulness way, including preservative games, encouraging regulation and communication by using facial expression, tone of voice and affect or imitating the child’s actions. In occupational therapy, we use the DIR floortimemethodology to promote the child’s participation in daily activities, play and motor skills while addressing and responding to sensory regulation and communication difficulties. This ‘face-to-face’ mode of intervention consists of pre-planned, semi-spontaneous play sessions in which relationships are built, and functional capacities such as self-regulation, two-way communication, social and emotional engagement, complex thinking and problem-solving are developed. On a table at the side of the room, we placed three transparent cylindrical containers with various threading elements inside and near them a basket with stickers. A barrel and a rocking bowl were placed on the floor in the middle of the room. On the other side of the room, we placed two storage boxes on a mattress, containing cars, dolls and utensils. The occupational therapists were instructed to sit near each station and avoid moving around in order to prevent emotional ‘flooding’ and to support the participants’ regulation. The children were allowed to approach the play stations according to their own preference. To help the children to participate in play, the occupational therapist demonstrated games, for example, tossing balls into the barrel or threading beads from the containers, encouraging the children to join, yet allowing them to choose an activity of their own choice. Because there were up to four children in a group, each occupational therapist played with one or two children at a time, whereas some children played on their own for up to 5 min, and then a therapist approached them. In certain instances, when the therapist noticed that a child was not interested in the stations, additional items were introduced including blocks, cons or scotch balls, depending on the cooperation and engagement in mutual activity. | The subjects underwent a weekly 45-min session of motor skill training for 8 consecutive weeks. The activities involved in the individual training and group training were essentially the same. A variety of functional tasks and exercises was designed to address common motor difficulties faced by children with DCD, such as agility, balance, core stability, and movement coordination. The same exercises were applied to both groups in each treatment session. As the training progressed, the motor tasks were adapted to ensure successful execution of the tasks while providing an adequate challenge to the child’s motor abilities. To maintain the consistency of the treatment approach, both the group-based and individual-based training were conducted by the same physiotherapist, who had many years of experience in paediatric rehabilitation. Each subject was given home exercises to reinforce what had been learned at each session and to increase the exercise frequency. The home exercises were selected from among those used in face-to-face training sessions, and were intended to address the specific areas of motor difficulty experienced by each child. The children were instructed to perform these exercises on a daily basis within the study period. The parents were provided with clear written instructions, and were asked to coach or assist their children in performing the home exercises. The exercise programme was designed to take approximately 20 min to complete. | The content and format were identical for both Indiv-rehab and Grp-rehab, involving 12 supervised sessions (twice weekly for 6 weeks) that combined discussion on specific topics regarding self-management and coping, etc., with an individualized, progressive exercise regimen. To ensure consistency in content and delivery, the same experienced physiotherapist devised, supervised, and progressed all sessions for all participants (NW). | A standardized voice therapy protocol was followed. Each therapy session lasted 30 minutes. Each therapist was present during the therapy sessions.The voice therapy sessions were designed to run over 6 weeks, either individually or as a group. Participants were randomly divided into two groups: receiving either individual voice therapy (eight participants) or group voice therapy (eight participants). | The treatment protocol was carried out as outpatient activities under the supervision of the same physical therapist that carried out the evaluation. The treatment for the GT and IT groups consisted of 12 sessions, with two 1 h sessions per week and total of 6 weeks of treatment. Both groups were taught to contract the pelvic floor muscles correctly, and the proper contractions were confirmed by vaginal palpation. The subjects of GT group were divided into groups of 8–10 people for treatment. During the sessions, volunteers received instructions about anatomy of the pelvic floor muscles and continence mechanisms and carried out exercises to strengthen the pelvic floor muscles in supine, sitting and standing positions. The difficulty degree progressed according to the positions adopted, increasing the number of repetitions and time of sustained contraction. An average of 100 contractions were performed per session, with phasic contractions, lasting three seconds with six seconds of rest, and tonic contractions, lasting 5–10 s followed by 10–20 s of rest [18]. To minimize the muscle fatigue, the resting time was rigidly observed in all sessions and the time of sustained contraction was slowly increased. At the beginning of the individual treatment, the time of sustained contraction was the same recorded for each woman during the initial evaluation. For the GT group, the mean time of the group was considered as the time of sustained contraction. For both groups, the time of sustained contraction was increased by 1 s per week up to 10 s. | Individual-based task-oriented circuit training (ITCT) (group II). Group II performed the same task done by Group III under the supervision of one physiotherapist with more than 10 years of physiotherapy experience. | Individual mirror therapy: Mirror therapy for the upper limb using an individual therapy protocol (i.e. one therapist treated one patient). In the first treatment session, patients were informed about mirror therapy and the principles of therapy in a single setting. Mirror therapy was minimally standardized as follows: both arms of the patient were placed on a table. A mirror (50 cm × 50 cm) was positioned between the two arms. The reflecting side of the mirror was adjusted to the non-affected arm. Patients were instructed to move both arms while looking in the mirror. The affected arm should be moved as well as possible. In the first week isolated movements of fingers, wrist, lower arm, elbow and shoulder joints were performed in all degrees of freedom. In each direction up to 50 repetitions per series and up to four series were possible. In the second and third week additional movements were used. Therapists were instructed to include object-related movements, such as putting a ball or bigger squares in different directions, moving sticks or wipe-like movements with a cloth. Type of movements, repetitions and series were adapted by therapists according to patients’ abilities. | The instructions and session intensity of the NBLI were similar for both groups, except for their presentation. Both groups also received CST sessions by their clinical practitioners concurrent to the intervention. |
| **Outcomes** | **Measured primary outcomes** | Gait speed | 1. strenghts performance 2. jump performance Testing consisted of a free weight full back squat 1-RM, and three jumping protocols including countermovement (CMJ), static-squat (SSJ), and standing-broad jumps (SBJ). | 1. amount of use / quality of movement 2. motor function We used the Chilean versions of the Motor Activity Log- 30 (MAL-30) and the Action Research Arm Test (ARAT) to evaluate daily function and motor function. | 1. Waiting days 2. Nonattendance 3. Staff burden of all therapists | 1. Motor performance, 2. Home exercise compliance 3. Parental satisfaction | Physical fuction | 1. maximum phonation time (MPT) (objective voice assessment), 2. s/z ratio (objective voice assessment), 3. F0 4. shimmer 5. jitter 6. NHR 7. Children’s perception of their voice disorder (subjective voice assessment) | Urinary loss | Gait abilities in patients after CTCT | Arm function on an impairment level and on a functional level | 1. Productivity of spoken narrative 2. Complexity of spoken narrative 3. Accuracy of spoken narrative 4. Story grammar of spoken narrative |
|  | **Data collection instruments (primary outcomes)** | 1. 6-minute walk test 2. 10-meter walk test Gait speed for each participant was calculated as the ratio between the walked distance and the time unit, and it was measured in meters/seconds. For each subject, all assessment sessions were performed in the morning, by the same person and all tests were performed in the same order, to control for variations in performance because of the medication cycle. All assessments were conducted in the “on” state for the subjects experiencing motor fluctuations. | Testing consisted of  1. a free weight full back squat 1-RM 2. three jumping protocols including countermovement (CMJ), static-squat (SSJ), and standing-broad jumps (SBJ). All testing and training took place at the same venue, under the direct supervision of the lead investigator, at the same time of the day (±1 hour) for each participant, and under consistent environmental conditions (~20°C). All jump variables were calculated using a force plate (Kistler, Winterthur, Switzerland; 1500 Hz) logged via Cortex software (Motion Analysis Corporation, CA, USA) and analysed using a custom MatLab script (MathWorks, MA, USA). Vertical jump height (CMJ, SSJ) and horizontal jump distance (SBJ) were calculated using take-off velocity of the centre of mass (COM) that was determined using the impulse-momentum relationship. Jump height was defined as the maximum estimated vertical displacement of the COM from take off, and distance as the estimated horizontal displacement of the COM between take-off and when vertical displacement of the COM was zero on the jump descent. This approach removed the effect of landing technique on jump performance. For the back squat, 1-RM and velocity profiling were established following an innovative progressive loading assessment, completed twice prior to the initiation of training (95% limits of agreement [LOA], coefficient of variation [CV], and intraclass correlation coefficient [ICC2,1] between visits. | 1. Chilean versions of the Motor Activity Log-30 (MAL-30)  2. the Action Research Arm Test (ARAT) MAL-30 provides a measure of a patient’s perception of real-world use of the hemiparetic upper extremity (HUE) in 30 relevant daily activities. Patients are asked to rate how much (amount of use or AOU scale) and how well (Quality of Movement scale) their HUE was used to accomplish each of the activities on a five-point response scale. Following research recommendations, only AOU scores were included in this study (Park et al., 2008). AOU scores range from 0 to 5; higher scores indicate greater amount of HUE use (Taub et al., 2006). ARAT assesses the upper extremity motor functional limitations in different tasks involving grasp, grip, pinch, and gross movement. Each of its 19 items is rated as follows (Yozbatiran et al., 2007): (1) 0= patient could not perform any part of the test within 60 s. (2) 1= patient could only partially perform the test within 60 s. (3) 2= patient could complete the test but took a considerably long time (5–60 s). (4) 3= patient performed the test normally within 5 s. Total scores range from 0 to 57; higher scores indicate normal functional performance. | 1. Waiting days defined as the number of waiting days from the date of recommendation for occupational therapy to the beginning of participation in an individual or group session. 2. Nonattendance defined as absence from a session for a variety of reasons. 3. Staff burden of all therapists was evaluated using a self-reported work satisfaction questionnaire at the commencement of the study and 5 months after it ended. The 11 variables included satisfaction with working conditions, people in the environment and general satisfaction. The items were rated on a 7-point scale that ranged from 1 (Very dissatisfied) to 7 (Very satisfied), based on Bowling and Hammond (2008). | 1. MABC (14) within one week of the initiation of the intervention and again within one week of the termination of the 8-week training programme. 2. A logbook was provided to parents for them to record on a daily basis (except for the days on which the face-to-face training sessions took place) whether their child had carried out the home exercise programme over the 8-week study period. The home exercise compliance rate (% days) was calculated using the following formula: (number of days of home exercise participation) × 100%/ (total number of days). 3. A parental satisfaction questionnaire was used to gather information on the perceived benefits of the motor skill training programmes (Table I). The questionnaire consisted of 2 parts, with part I (5 questions) and part II (8 questions) addressing the perceived benefits of the programme for the parents themselves and for the children, respectively. | Likert version of the Western Ontario and McMaster Universities Osteoarthritis Index (WOMAC). This questionnaire produces a total score (WOMAC-total, 0–96 points), with subscores for physical functioning (WOMAC-func, 0–68 points), pain (WOMAC-pain, 0–20 points), and stiffness (0–8). | 1. sustained /i/ phonation was used. Participants were asked to phonate /i/ for as long as they could. It was recorded and calculated for all of the participants. 2. all of the participants were asked to sustain /s/ and /z/ phonemes three times and the longest duration was used to calculate the s/z ratio. 3-6. All recordings were made using a Samson Go Mic in a quiet room with the microphone positioned 10 cm from the lips of the participants. Acoustics analysis was completed using sustained /i/ phonation. Analysis of the voice samples was conducted by 2 SLTs using PRAAT.  7. The Turkish Children’s Voice Handicap Index- 10 (Tr-CVHI-10) (10 questions) | The 1-h pad test was carried out to evaluate urinary loss according to the protocol proposed by Abrams et al. [13]. The volunteers were instructed to wear a pad previously weighed on a precision balance Denver APX200 (precision of 0.0001 g, Denver Instrument, Denver, USA) and then drink 500 ml of water. After 30 min they started performing a series of provocative exercises: walking for 10 min, including stair climbing equivalent to one flight up and down; standing up from sitting, 10 times; coughing vigorously, 10 times; running on the spot for 1 min; bending to pick up small object from floor, five times; and washing hands in running water for 1 min. At the end of 1 h, the pad was removed, reweighed and the urinary loss was calculated. The ICC and the SEM for this variable were 0.99 and 0.45 g. | A GAITRite (CIR Systems Inc., Sparks, NJ, USA): to measure temporal and spatial variables to test gait abilities. The subjects walked from a place 2 m away from the electronic walking mat start point to a place 2 m away from the end position to reduce the acceleration and deceleration effect6). This study measured velocity, cadence, stance phase symmetry profile, swing phase symmetry profile, and step length symmetry profile. To determine the walking endurance, a two To determine the walking endurance, a two-minute walking test (2MWT) was conducted7). A 50 cm-high cylinder was placed 1 m and 9 m away from the start point, and the subjects walked with a comfortable velocity around the two cylinders for the 2MWT8). A researcher recorded the number of times that each subject walked around the cylinders, which was subsequently converted into a distance. He then added a distance from the heel of the subject to the cylinder at the completion of the 2MWT to calculate the final walking distance. | On an impairment level assessed with the motor score of the Fugl-Meyer Test, on a functional level with the Action Research Arm Test. | Persian version of the Language Sample Analysis (LSA) test 1. number of total words (NTW), number of cohesive markers (NCM), and number of total clauses (NTC) 2. the average T-unit length (ATL) and subordination index (SI) 3. semantic/syntactic accuracy 4. number of story grammars (NGS) |
|  | **Measured secondary outcomes** | NR | NR | NR | 1. adaptive behaviour 2. quality of life 3. fine motor skills | NR | 1. pain 2. objective functional performance 3. changes in health beliefs and self-efficacy 4. Anxiety and Depression 5. health-related quality of life 6. health status 7. quadriceps strength 8. quadriceps voluntary activation | NR | 1. quality of life 2. pelvic floor muscle pressure, 3. pelvic floor muscle strength 4. satisfaction with treatment | NR | 1. basic activities of daily living 2. self-reported influence of stroke-related functioning on quality of life 3. somatosensory, pain and range of motion sections 4. changes in resistance to passive movement of finger and wrist flexors 5. visuospatial neglect | NR |
|  | **Data collection instruments (secondary outcomes)** | NR | NR | NR | 1. To assess adaptive behaviour, we used the Adaptive Behaviour Assessment System (ABAS) questionnaire, which included the composite score of conceptual, practical and social functioning measurements and the total composite score (Harrison & Oakland, 2008).  2. The participants’ quality of life was assessed using the Paediatric Quality of Life Inventory (PDSQL) (Varni et al., 2001).  3. Fine motor skills were evaluated by a certified occupational therapist using the Peabody Developmental Motor Scales Second Edition (PDMS-II) subtests numbers 5 and 6, which examined types of fine grips and visual-motor integration (Folio & Fewell, 2000). | NR | 1. WOMAC-pain 2. AFPT ((aggregated functional performance time of 4 common activities of daily living [AFPT]) (29) 3. exercise health beliefs and self-efficacy questionnaire (ExBeliefs) 4. Hospital Anxiety and Depression Scale [HADS] 5.condition-specific patient preference health-related quality of life questionnaire (McMaster Toronto Arthritis [MACTAR]) 6. EuroQol EuroQol) (32) converted into qualityadjusted life years based on utility weights collected in a UK general population sample (33) 7. quadriceps maximum voluntary contraction 8. quadriceps voluntary activation | NR | 1. For the assessment of quality of life, the King’s Health Questionnare (KHQ) (30 questions, divided into nine individually scored domains. These domains (ICC; SEM) are: general health (0.79; 8.69), incontinence impact (0.82; 13.33), limitations of daily activities (0.97; 6.50), physical limitations (0.93; 8.43), social limitations (0.96; 6.21), personal relationships (0.76; 7.31), emotions (0.96; 4.05), sleep and disposition (0.85; 11.75) and gravity (0.91; 7.60).)  2. To evaluate the pelvic floor muscle pressure, a Perina Stim device (Quark Medical Products, Piracicaba, Brazil) was used, graded from 0 to 60 cmH2O and supplied with a vaginal probe. The volunteers were positioned in supine, with hip and knee flexion. The vaginal probe was inserted in the vagina and the device was calibrated. Then, the volunteers were asked to perform three 3-s maximum perceived effort contractions of pelvic floor muscles. The volunteers were instructed not to use abdominal, gluteal and/ or hip adductor muscles during the contractions [16]. The ICC and SEM were 0.97 and 0.53 cmH2O, respectively. 3. The assessment of pelvic floor muscle strength by digital palpation was carried out using the PERFECT scheme. The volunteers were positioned in supine with hip and knee flexion. In this position, the evaluator introduced two fingers up to one third of the vagina. The volunteer was then instructed to lift and squeeze the pelvic floor muscle as hard as possible. The strength measured on the 6-point Modified Oxford Scale [17]. The ICC and SEM for this variable were 0.84 and 0.20. 4. In the end of treatment, the volunteers were questioned regarding their satisfaction with treatment | NR | 1. Barthel Index for evaluating basic activities of daily living 2. Stroke Impact Scale to detect self-reported influence of stroke-related functioning on quality of life 3. the somatosensory, pain and range of motion sections of the Fugl-Meyer Test - arm section 4. the modified Ashworth Scale to detect changes in resistance to passive movement of finger and wrist flexors 5. Star Cancellation Test for assessing visuospatial neglect | NR |
|  | **Measurement time points** | All patients were evaluated at the beginning and at the end of the physical therapy program | Before and after 6 weeks of resistance training | 1 week before intervention, two trained unbiased evaluators performed a pretreatment evaluation and, subsequently, under the same conditions, a post-treatment and a follow-up (6 months) evaluation of the dependent variables. | At the first evaluation and after the end of the intervention | 1. Motor performance was evaluated using the MABC (14) within one week of the initiation of the intervention and again within one week of the termination of the 8-week training programme. 2. A logbook was provided to parents for them to record on a daily basis (except for the days on which the face-to-face training sessions took place) whether their child had carried out the home exercise programme over the 8-week study period. 3. The parents were given the questionnaire at the end of the final training session. | All outcomes were assessed at baseline, immediately after completion of the intervention or recruitment to the usual primary care arm (6-week assessment), and 6 months after completion of rehabilitation | Once before, once after completing the therapy sessions (after the third month) | Before and after treatment | Measurements were conducted before and after the intervention | Measurements were taken at baseline and after the intervention | Before intervention (T0), immediately after intervention (T1), two months after intervention (T2) |
|  | **Effect measures** | Quantitative data were tested for normality of distribution and expressed as mean±standard deviation or median and interquartile range (IQR), whenever appropriate. Qualitative data were expressed as frequency and percent. Comparison between groups was performed using the Student t-test, Mann-Whitney test or chi-square test, whenever appropriate. Correlations between two groups were tested using the Spearman’s rho correlation coefficient. A p-value <0.05 was considered statistically significant. | For all variables, values are presented as means ± SD. Data analyses were completed using SPSS 22.0 (Chicago, IL, USA), with the alpha level for significance set at α = 0.05. Appropriate statistical assumptions of normality and sphericity were confirmed prior to running any analyses on the data. Independent sample t-tests were completed to examine the pre-training intergroup differences, as well as post-training total volume relationship. Paired samples t-tests were completed to examine the intra-group percentage difference pre- to posttraining. Two-way mixed (between-within) ANOVA, with Bonferroni post-hoc comparisons, using one inter-factor (ILVP vs. GLVP) and one intra-factor (pre- vs. post-training), were conducted to examine the differences across the back squat and all jump protocols between groups. In addition, effect sizes (ES) were calculated according to Hedge’s g, accounting for sample size. ES were classified as small (g = 0.21–0.59), moderate (g = 0.60–1.19), large (g = 1.20–1.99), and very large (g ≥ 2.0). | We carried out analyses of covariance (ANCOVAs) to determine whether post-test and follow-up scores were significantly different between the two treatment modalities once pretest scores were held constant. For each of the outcome variables, we carried out two one-way between-patient ANCOVAs. In the first analysis, we used the post-test scores as the dependent variable and the pretest scores as the covariate. The treatment modality, that is, the exposure of patients to group or individual CIMT was the independent variable. In the second analysis, to evaluate the stability of changes, we repeated the ANCOVAs using, this time, the follow-up scores as the dependent variable and the pretest scores as the covariate. Before ANCOVAs, the homogeneity-ofslope assumption was examined. | Categorical variables were summarised using number and percentage and examined for statistical differences using Fisher’s exact test. Continuous variables were summarised using mean and standard deviation and were examined for statistical differences using Wilcoxon’s test for changes within each study group and the Mann–Whitney test for differences between groups. Changes in work satisfaction score were tested using a one sample t-test. Cohen’s d effect size within each study group and between groups was calculated. A p-value <0.05 was considered statistically significant. | Non-parametric statistic analysis was used due to the relatively small sample size. To ensure that the baseline parameters were comparable between the 2 groups, the Mann-Whitney U test (for age and total impairment score) and the χ2 test (for the proportion of boys and girls) were used. To assess the effect of each type of intervention on motor performance (the within-group effect), Wilcoxon-signed rank tests were used to compare the MABC total impairment score and the subtest scores before and after the intervention for each group. The Mann-Whitney U tests were then used to compare the change in total impairment score and subtest scores (the post-test score minus the pre-test score) between the 2 groups (the between-group effect). As a higher total impairment score indicates more severe motor impairment, a more negative change in total impairment score represents a greater improvement in motor abilities. The Mann-Whitney U test was also used to compare the home exercise compliance rate and parental satisfaction scores of the 2 groups. The Spearman’s rho correlation coefficient was used to explore whether the change in total impairment score was associated with the baseline total impairment score or home exercise compliance rate. All of the statistical analyses were performed using the Statistical Package for the Social Sciences (SPSS Inc., Chicago, IL, USA) software program version 16.0. The level of significance was set at 0.05 (2-tailed). In addition, post-hoc power analysis was performed using the G*Power statistical software (Faul & Erdfelder, Bonn University, Germany, 1992). | Statistical significance was set at P less than 0.05. Outcomes for the 3 trial arms are presented as the mean SD or median (range), and standardized effect sizes (95% confidence intervals [95% CIs]) were calculated from the difference between the mean outcome at 6 months for one group minus the mean outcome at 6 months for another divided by the SD of the mean outcomes adjusted for baseline outcomes. | In this study, the s/z ratio, MPT and acoustic parameters such as F0, shimmer, jitter and NHR were selected as dependent variables; the therapy receiving format − either group or individual − was an independent variable.Data analyses were conducted using the SPSS 22.0 package. As the normality assumption was violated, a nonparametric Wilcoxon test was applied (P < 0.05). | To test the normal distribution of data in each group, the Shapiro–Wilk test was used and to verify the homogeneity of the groups a Kruskal–Wallis test was applied. Because the data were not normally distributed, the intragroup analysis was carried out using Wilcoxon nonparametric test. For the intergroup analysis the Kruskal–Wallis test was used. Pairwise comparisons were made with the Mann–Whitney test to compare the groups. Differences were considered significant when the p value was <0.05. To measure the clinical significance of the data, the effect size and the confidence interval were calculated. The effect sizes were considered mild if values were smaller than 0.20; moderate if values were between 0.25 and 0.75, and large when values were over 0.80 [19]. | The statistical significance level α was set to 0.05 | Differences between baseline characteristics were tested using an analysis of variance (ANOVA) for continuous variables, the Kruskal–Wallis test for ordinal scaled variables and the chi-square test for dichotomous variables. For testing group differences between baseline and post-intervention data we used an ANOVA to compare change scores between pre- and post-intervention data for continuous variables and the Kruskal–Wallis test for ordinal scaled variables. If significant results were found we underwent a post-hoc comparison. All tests were conducted on a 5% significance level. An intention-to-treat analysis was performed using the last observation carried forward method. | In the current study, continuous data is presented as mean ± SD and discontinuous data is presented as frequency (or percentage of frequency). After checking the normality of the distribution of the continuous data using the one-sample Kolmogorov-Smirnov test and the homogeneity of variance using Mauchley's test, one-way analysis of variances (ANOVA) was used to compare the mean values of the dependent variables at three points during treatment (T0, T1, T2) between the groups of CI children. Following that, a (3 groups: NBLI(G) vs. NBLI(I) vs. CST)×(3 time points of treatments at: T0, T1, T3) two-way repeated measures analysis of variances (RM-ANOVA) test was used to compare the mean values of the dependent variables of spoken narrative within-groups. The main effects as well as the interaction effects of the independent variables on the outcomes were computed. If a main effect for either the time points or groups was observed, the post hoc adjusted Bonferroni test was used to determine the differences within factors for the continuous data. If homogeneity of variances was not observed for continuous data by Mauchley's test, Greenhouse-Geisser estimates of sphericity was used to correct the degrees of freedom. The level of significance was set at p≤0.05 for this data. Given that the NTW, NCM, NTC and number of story grammars (NSG) were discontinuous data, and had not normal distribution based on Kolmogorov-Smirnov test the nonparametric Friedman's test and Kruskal-Wallis's test were used to determine the within-between group differences. The post hoc Wilcoxon signed rank test and the adjusted Bonferroni method was applied for paired differences within factors (time points of treatments and groups). The level of significance was set at p≤0.01 for this data. In order to assess the importance of differences caused by the time points of treatments and group factors, both effect sizes (η2) and statistical power were used. |
|  | **Primary outcomes** | There were no differences between the 2 groups regarding initial gait speed, as measured by the 10-meter or 6-minute walk tests (all p>0.05). 1. There was an increase in both groups regarding gait speed after treatment (all p<0.05), as measured by the two instruments, except for the values obtained by the 6-minute walk test in the IPT group, which did not improve significantly (p=0.07) (Table II). The 6-minute walk test scores after treatment showed significantly higher values of gait speed in the group therapy compared to individual therapy (p=0.002) (Table III).  2. In the group therapy, the 10-meter walk test at normal speed showed higher gait speeds than in the individual therapy group, but it did not reach statistical significance (p=0.06). The 10-meter walk test at maximum speed showed no statistically significant difference between the treatment groups (p=0.72). | 1. Strength assesments: Training resulted in significant increases in back squat 1-RM for the ILVP and GLVP group (p < 0.01; 9.7% and 7.2%, respectively), with no group-by-time interaction identified between training groups (F(1,17) = 3.97 p = 0.06).  2. Jump assessments: Significant increases in CMJ, SSJ, and SBJ performance were noted for the ILVP group (p < 0.01; % increase: CMJ: 6.6%; SSJ: 4.6%; SBJ: 6.7%), and CMJ and SSJ only for the GLVP group (p < 0.05; both 4.3%; Figure 1). No significant group by time interactions was reported between the groups (CMJ: F(1,17) = 2.50 p = 0.13; SSJ: F(1,17) = 0.15 p = 0.71; SBJ: F(1,17) = 3.49 p = 0.08). | 1. MAL mean scores increased from the pretest to the posttest by 1.30 points for the group CIMT and by 1.18 points for the individual CIMT. We carried out an ANCOVA to verify whether post-test scores differed significantly by treatment modalities when holding pretest scores constant. A preliminary analysis assessing the homogeneity-of-slopes assumption showed that the relationship between the covariate and the dependent variable did not differ significantly in each treatment modality, F(1, 32) =0.180, P= 0.674. The ANCOVA indicated a significant difference between the treatment conditions favoring the group therapy, after controlling for the pretest scores, F(1, 31) =21.55, P < 0.001, η2 p= 0.57. 2. The mean scores of ARAT increased from the pretest to the post-test by 4.95 points for the CIMT group and by 4.94 points for the individual CIMT group. An ANCOVA indicated a significant difference between the treatment conditions favoring the group therapy, after controlling for the pretest scores, F(1, 31)=4,72, P=0.016, η2 p=0.22. Previously, the homogeneity-of-slopes assumption was fulfilled, F(1, 32)=2.068, P=0.160. The means of ARAT showed a small variation from the post-test to the follow-up for both treatment conditions. An ANCOVA with the follow-up scores as the dependent variable and the pretest scores as the covariate showed a significant difference in favor of the group therapy, F(1, 31)=3.69.44, P = 0.036, η2 p= 0.18. Again, the homogeneity-of-slopes supposition was achieved previously, F(1, 32) =2.143, P= 0.153. By controlling the preintervention evaluations, analyses of covariance indicated that both dependent variables presented significant differences in favor of the group therapy at both the postintervention evaluation and the follow-up evaluations. Both types of intervention generated increases in the function and use of the upper extremity, with these increases being higher in the group therapy. The effects of the group therapy modality were maintained 6 months after the intervention ended. | 1. Participants waited fewer days to commence group compared to individual occupational therapy (52.4 ± 28.1 vs. 108.8 ± 48.0 days, p < 0.01).  2. The mean number of nonattendance was similar in both interventions (3.2 ± 2.82 vs. 2 ± 1.76, p > 0.05).  3. No differences were found between therapists’ work satisfaction scores at the beginning of the study and 5 months thereafter (6.1 ± 0.4 vs. 6.07 ± 0.49, p > 0.05). | 1. In the pooled data, a significant decrease in the total impairment score was found in both the group-based training group (mean –4.4 (SD 5.0), p = 0.003) and the individualbased training group following the intervention (mean –5.2 (SD#5.1), p = 0.016), indicating an improvement in motor performance. The change in total impairment score was not significantly different between the 2 groups (p = 0.379). Six of the children in each group attained a MABC score of greater than the 15th percentile (denoting no motor difficulty) post-intervention. Although the MABC total impairment score decreased for most of the children following individualbased training, it seems that in the group-based training group, those who had a higher initial total impairment score (more severe impairment) tended to experience a greater improvement than those who had milder motor impairment (Fig. 2). Indeed, the association between the MABC total impairment score at baseline and the change in total MABC score was not significant for the individual-based training group (p = –0.082, p = 0.811), but was marginally significant for the group-based training group (p = 0.542, p = 0.069). In examining the 3 cluster scores of the MABC (manual dexterity, ball skills, static/dynamic balance), it was found that both treatment groups showed significant improvement in manual dexterity (p < 0.05). In addition, the children in the individual-based training group showed a significant improvement in static/dynamic balance over time (p = 0.040). However, there was no significant between-group difference in the change in scores for the 3 subtests, indicating that the improvement in these 3 clusters was similar following the individual-based and group-based training. 2. Home exercise compliance: Home exercise compliance was similar between the 2 groups (individual-based: mean 62.2% (SD 29.3%); group-based: mean 50.1% (SD 23.7%), p = 0.288). 3. Parental satisfaction: 0.915). In terms of parental satisfaction (Table I), there was no significant between-group difference in the satisfaction score for each individual item (p > 0.200) or in the overall satisfaction score (p = 0.379). | 1. functioning improved (usual care 25.9, 95% CI 23.4, 28.3; rehabilitation 20.0, 95% CI 18.3, 21.7; Indiv-rehab 19.8, 95% CI 17.6, 22.0; Grp-rehab 20.2, 95% CI 17.6, 22.9). At the primary end point, 6 months after completing the program, some of these improvements had been lost but  2. WOMAC-func differed significantly across the 3 arms (joint Wald’s test, P 0.04). Mean WOMAC-func scores for the Indiv-rehab and Grprehab arms were significantly different from usual care (difference in WOMAC-func: Indiv-rehab 3.53, 95% CI6.52, 0.55, P 0.04; Grp-rehab 3.16, 95% CI 6.55,0.12, P 0.04) but not from each other (Indiv-rehab 21.5, 95% CI 19.3, 23.6; Grp-rehab 21.8, 95% CI 19.6, 24.0).  3. NNT was 7 (95% CI 4, 27; calculation of NNTs from inversion of the relative risk causes asymmetric 95% CI). | 1. MPT was compared between groups and within groups. Although MPT was longer in all the groups, there was no statistical difference within and between the groups (P = 0,945). There was no significant differences between the p-VHI scores of the individual therapy participants (P = 0,249) and group therapy participants (P = 0,528). No significant difference was found inter-group regarding pre and post therapy scores (P = 0,234). 2. There was a statistically significant difference (P = 0,014) between the s/z ratios of the inter groups. When this difference was analyzed, it was revealed that those who took part in the individual therapy had a ratio that was closer to one compared to the group therapy. 3-4. There was no statistical difference between F0 (P = 0.628) and shimmer (P = 0.534) scores inter group pre and post therapy. 5. There was no statistically significant difference between jitter of the group therapy participants (P = 0,058) and individual therapy participants (P = 0.398). However, there was a statistically significant difference between inter-groups (P = 0,005). When this discrepancy was studied, it was revealed that jitter scores were better in group therapy participants (Table 2) 6. There was a statistically significant difference between NHR of the individual therapy participants (P = 0,018), but no significant difference was found in group therapy participants (P = 0.917). There was a statistically significant difference between inter-groups (P = 0,022). When this discrepancy was studied, it was revealed that NHR scores were better in group therapy participants.  7. There was no significant differences between the p-VHI scores of the individual therapy participants (P = 0,249) and group therapy participants (P = 0,528). No significant difference was found inter-group regarding pre and post therapy scores (P = 0,234). | There was a significant reduction in urinary loss measured by pad test in the IT group after treatment. In CG and GT groups there were no significant differences in this variable, although it has been observed a trend for the reduction in urinary loss for the latter. In the intergroup analysis, there was a significant difference only after treatment between GT group and CG (p < 0.0001; effect size 0.91; 95% confidence interval from 0.56 to 5.80) as well as between IT group and CG (p < 0.0001; effect size 0.90; 95% confidence interval from 0.54 to 5.84). | To determine the difference in gait velocity between groups, ANCOVA was conducted, and the results showed a statistically significant difference (p < 0.05). The Bonferroni post-test results showed (...), while no significant difference was shown between Groups II and III. To compare the difference in cadence between groups, ANCOVA was conducted, and the results showed a statistically significant difference (p < 0.05). After the Bonferroni post test, the results showed (...) No significant difference was shown between (...) Groups II and III (Table 1). To compare the difference in symmetry profiles in the stance and swing phases between groups, ANCOVA was conducted, and the results showed no statistically significant difference. To compare the difference in step length symmetry profiles between groups, ANCOVA was conducted, and the results showed no statistically significant difference. To determine the difference in the 2MWT between groups, ANCOVA was conducted, and the results showed a statistically significant difference (p < 0.01). Using the Bonferroni post test, a significant difference was found between (...). No significant difference was found between Groups II and III (Table 1). | After the intervention, the Action Research Arm Test score of all participants increased significantly over time (F = 10.2, P = 0.002). There were no significant group differences (F = 0.8, P = 0.44). Fugl-Meyer Test motor scores for the upper limb for all participants increased over time (F = 18.0, P < 0.001) with no differences between groups (F = 0.4, P = 0.71). Regarding the Fugl-Meyer Test subscores for the arm, hand and fingers, no significant interactions between groups were detected. | 1. The withingroup comparisons show that both CI children groups who received NBLI had significantly higher median scores for NTW, NCM and NTC at T1 and T2 than at T0 (p≤0.008), but the median scores for the group that received CST had not significantly increased (p≥0.110). Betweengroups comparisons showed that all components of productivity of spoken narrative for the groups who received NBLI were significantly higher than for the group that received CST at T1 and T2 (p≤0.008). 2. The within-group comparisons showed that two groups who received NBLI had significant improvement in ATL and SI scores at T1 and T2 compared to T0 (p≤0.01). The mean ATL and SI scores at T1 and T2 were similar, with the exception of the ATL score for group 1 (p=0.008), which increased significantly. There were no significant differences between the groups who received individuallydelivered NBLI and who had the group therapy form of NBLI. There also was no significant testing time×group interaction on ATL [F(4,66)=1.16, p=0.33, power=0.346] and SI [F(4,66)=1.82, p=0.31, power=0.248]. 3. Withingroup comparison revealed that all groups related spoken narrative with greater semantic/syntactic accuracy at T1 and T2 than at T0 (p≤0.04). The mean value of accuracy of performance of spoken narrative at T2 showed significant increases over T1 in all groups except the group 3 (p=0.168).There were no significant differences between the groups that received individually-delivered NBLI and the group therapy form of NBLI. There also was no significant effect for testing time×group interaction on the accuracy of performance of spoken narrative [F(4,66)=0.88, p=0.46, power=0.235]. 4. Within-group comparisons showed that all three groups performed significantly better on the number of story grammar elements at T1 and T2 than at T0. While the median NSG score at T2 increased significantly compared to T1 in the groups that received NBLI (p≤0.007), no difference was found between T2 compared to T1 in the group that received CST. Between-groups comparison showed that the median NSG score of spoken narrative in the groups that received NBLI were significantly higher than for the group that received CST at T2 (p=0.008), but no difference was seen between groups at T1 (p=0.11). |
|  | **Secondary outcomes** | NR | NR | NR | The clinical outcomes of both group and individual occupational therapy at the end of the study are shown in Table 3. There were no significant differences between baseline and follow-up outcomes within both group and individual therapy. In addition, there were no significant differences in percentage changes between individual and group therapy outcomes for  1. adaptive score (6.0 ± 16.0 vs. 4.5 ± 17.9, p > 0.05) 2. quality of life, (1.3 ± 20.9 vs. 18.8 ± 24.4, p > 0.05) 3. fine motor skills (13.7 ± 36.1 vs. 15.1 ± 41.5, p > 0.05). | NR | A similar pattern of results was found for the secondary outcomes (Table 2) and effect sizes (Table 3): there were differences between rehabilitation and usual care, but no difference between Indiv-rehab and Grp-rehab. | NR | 1. In evaluation of quality of life, a significant reduction of scores in the incontinence impact, limitations of daily activities and gravity domains was observed for both GT and IT groups after treatment, as well as a significant difference only for the GT group in the sleep and disposition domain. In the physical limitations and emotions domains, significant differences after treatment were found only in IT group. In the general health, social limitations and personal relationships domains, no significant differences were observed for any of the treatment groups. In the intergroup analysis, significant differences were observed in incontinence impact, physical limitations and gravity domains (p < 0.01 for all variables) when comparing both treated groups and CG after treatment. In the domains of limitations of daily activities and emotions, significant differences (p = 0.03 and p = 0.002, respectively) were found only in GT group when compared to CG after treatment. Significant differences between GT and IT groups after treatment were only found in personal relationships and emotions domains (p = 0.02 and p = 0.03, respectively). For general health, social limitations and sleep and disposition domains, there were no differences among groups (Table 3). The effect size and 95% confidence interval for the KHQ domains were calculated and are shown in Table 4. 2. The pressure perineometry of the pelvic floor muscles was significantly increased in both GT and IT (...). The intergroup analysis showed statistical differences between GT group and CG (p = 0.0001; effect size 1.78; 95% confidence interval from 14.63 to 35.81) as well as between IT group and CG (p < 0.0001; effect size 1.87; 95% confidence interval from 15.98 to 37.26) only after treatment.  3. There was significant improvement in muscle strength as measured by the Oxford scale in GT and IT groups after treatment. The intergroup analysis verified statistical differences between GT group and CG (p < 0.0001; effect size 2.59; 95% confidence interval from 1.14 to 2.06) as well as between IT group and CG (p = 0.0008; effect size 1.63; 95% confidence interval from 0.68 to 1.84). For both variables,vthere were no differences between treated groups. 4. Regarding satisfaction with the received treatment, 13 out of 15 volunteers (86%) from each of the treatment groups reported positively in this matter; therefore, the two treatment groups had the same subjective satisfaction. There were no complaints of adverse effects due to treatment from either group. | NR | 1. The Barthel Index (...) no significant group differences were found (F = 0.4, P = 0.7).  2. The Stroke Impact Scale total score (...) but no significant group differences were detected (F = 0.3, P = 0.78).  3. Even the somatosensory subscore of the Fugl-Meyer Test (...) with no significant group interaction (F = 0.4, P = 0.68). The passive range of motion and pain scores for all participants (...) However, no group interactions were found (F = 1.5, P = 0.23; F = 0.4, P = 0.69).  4. Resistance against passive movement measured with the Modified Ashworth Scale (...). We detected a significant group difference between individual mirror therapy and the mirror therapy group intervention, indicating higher scores for individual mirror therapy after the intervention (P < 0.05). No significant group interaction was found for wrist flexors (P = 0.82) 5. Fourteen patients were found to have a visuospatial neglect using the Star Cancellation Test. Three patients receiving individual mirror therapy, five in the mirror therapy group intervention and six in the control group were included in the analysis. Patients receiving individual mirror therapy had an increase of the total score of 20.0 (14.5); those receiving group mirror therapy had an increase of 4.4 (6.7). In contrast, the score decreased by about –2.3 (5.2) for the six participants in the control group. We found a significant group interaction for the Star Cancellation Test score over time (F = 7.5, P = 0.009). Post-hoc analysis revealed a significant group difference between individual mirror therapy and the control group (P < 0.01) (Figure 2). No other group comparisons were significant. | NR |

Supplementary Table S4. Full-text screening and reasons for exclusion

| **Author** | **Year** | **Title** | **Inclusion/ Exclusion** | **Reason for exclusion** | **Source** |
| --- | --- | --- | --- | --- | --- |
| Abrahamsson et al. | 2018 | Effects of Voice Therapy: A Comparison Between Individual and Group Therapy | Exclusion | No randomization | Literature search |
| Ager et al. | 2019 | The Effectiveness of an Upper Extremity Neuromuscular Training Program on the Shoulder Function of Military Members With a Rotator Cuff Tendinopathy: A Pilot Randomized Controlled Trial | Exclusion | Interventions not comparable between groups | Literature search |
| Agger et al. | 2024 | Patient Preferences and Their Effects on Rehabilitation Outcomes: A Secondary Analysis of a Randomized Controlled Trial | Exclusion | Interventions not comparable between groups | Literature search |
| Alam et al. | 2018 | Comparison of Group Therapy versus Individual Therapy for Hyperfunctional Voice Disorders among Teachers | Exclusion | No randomization | Literature search |
| Aprile et al. | 2011 | Group rehabilitation versus individual rehabilitation following knee and hip replacement: a pilot study with randomized, single-blind, cross-over design | Exclusion | No randomization | Literature search |
| Arnott et al. | 2014 | Group Lidcombe Program Treatment for Early Stuttering: A Randomized Controlled Trial | Exclusion | No randomization | Literature search |
| Bech et al. | 2021 | The effect of group or individualised pelvic floor exercises with or without ultrasonography guidance for urinary incontinence in elderly women - A pilot study | Exclusion | Interventions not comparable between groups | Literature search |
| Borsati et al. | 2024 | Feasibility of a novel exercise program for patients with breast cancer offering different modalities and based on patient preference | Exclusion | No randomization | Literature search |
| Boyle et al. | 2007 | A randomised controlled trial and economic evaluation of direct versus indirect and individual versus group modes of speech and language therapy for children with primary language impairment | Exclusion | Interventions not comparable between groups | Literature search |
| Boyle et al. | 2009 | Direct versus indirect and individual versus group modes of language therapy for children with primary language impairment: principal outcomes from a randomized controlled trial and economic evaluation | Exclusion | Interventions not comparable between groups | Literature search |
| Brueggen et al. | 2017 | Cognitive Rehabilitation in Alzheimer’s Disease: A Controlled Intervention Trial | Exclusion | Interventions not comparable between groups | Literature search |
| Cacciari et al. | 2021 | Pelvic floor morphometrical and functional changes immediately after pelvic floor muscle training and at 1‐year follow‐up, in older incontinent women | Exclusion | Interventions not comparable between groups | Literature search |
| Cacciari et al. | 2022 | Group-based pelvic floor muscle training is a more cost-effective approach to treat urinary incontinence in older women: economic analysis of a randomised trial | Exclusion | Interventions not comparable between groups | Literature search |
| Carr et al. | 2005 | A randomized trial comparing a group exercise programme for back pain patients with individual physiotherapy in a severely deprived area | Exclusion | Interventions not comparable between groups | Review |
| Chown et al. | 2008 | A prospective study of patients with chronic back pain randomised to group exercise, physiotherapy or osteopathy | Exclusion | Interventions not comparable between groups | Literature search |
| Christiansen et al. | 2021 | Group-based exercise, individually supervised exercise and home-based exercise have similar clinical effects and cost-effectiveness in people with subacromial pain: a randomised trial | Exclusion | Interventions not comparable between groups | Literature search |
| Ciortea et al. | 2020 | Effects of group physical therapy on the walking speed in patients with Parkinson’s disease | Inclusion |  | Literature search |
| Collins et al. | 2013 | Hearing aid effectiveness after aural rehabilitation: individual versus group trial results | Exclusion | Interventions not comparable between groups | Review |
| Collis et al. | 2020 | Supporting parents of children aged 2–13 with toileting difficulties: Group-based workshops versus individual appointments | Exclusion | No randomization | Literature search |
| De Greef et al. | 2011 | Increasing Physical Activity in Belgian Type 2 Diabetes Patients: a Three-Arm Randomized Controlled Trial | Exclusion | Interventions not comparable between groups | Literature search |
| de Oliveira Camargo et al. | 2009 | Pelvic floor muscle training in female stress urinary incontinence: comparison between group training and individual treatment using PERFECT assessment scheme | Exclusion | Interventions not comparable between groups | Review |
| Dennis et al. | 2025 | Exploration of heterogeneity of treatment effects across exercise-based interventions for knee osteoarthritis | Exclusion | No randomization | Literature search |
| Dickson et al. | 2009 | Cost analysis of direct versus indirect and individual versus group modes of manualbased speech-and-language therapy for primary school-age children with primary language impairment | Exclusion | Interventions not comparable between groups | Literature search |
| Dorrell et al. | 2020 | Comparison of individual and group-based load-velocity profiling as a means to dictate training load over a 6-week strength and power intervention | Inclusion |  | Literature search |
| Doussoulin et al. | 2018 | Effects of modified constraint-induced movement therapy in the recovery of upper extremity function affected by a stroke: a single-blind randomized parallel trial-comparing group versus individual intervention | Inclusion |  | Literature search |
| Dumoulin et al. | 2020 | Group-Based vs Individual Pelvic Floor Muscle Training to Treat Urinary Incontinence in Older Women A Randomized Clinical Trial | Exclusion | Interventions not comparable between groups | Literature search |
| Dupuis et al. | 2024 | Group Physical Therapy Programs for Military Members With Musculoskeletal Disorders: A Pragmatic Randomized Controlled Trial | Exclusion | Interventions not comparable between groups | Literature search |
| Dupuis et al. | 2025 | Is Group-Based Physiotherapy a Cost-Effective Intervention Compared to Usual One-on-One Physiotherapy Care in the Management of Musculoskeletal Disorders in Active Military Personnel? An Economic Evaluation Alongside a Pragmatic Randomized Clinical Trial | Exclusion | Interventions not comparable between groups | Literature search |
| Eidsvåg et al. | 2019 | Individual Versus Small Group Treatment of Morphological Errors for Children With Developmental Language Disorder | Exclusion | No randomization | Literature search |
| Euler et al. | 2014 | The effectiveness of stuttering treatments in Germany | Exclusion | No randomization | Literature search |
| Fransen et al. | 2001 | Physical therapy is effective for patients with osteoarthritis of the knee: a randomized controlled clinical trial | Exclusion | Interventions not comparable between groups | Review |
| Freene et al. | 2015 | Longitudinal comparison of a physiotherapist-led, home-based and group-based program for increasing physical activity in community-dwelling middle-aged adults | Exclusion | No randomization | Literature search |
| Galea et al. | 2016 | Implementation of a technology-assisted programme to intensify upper limb rehabilitation in neurologically impaired participants: a prospective study | Exclusion | No randomization | Literature search |
| G. Gillon and B. Dodd | 1997 | Enhancing the phonological processing skills of children with specific reading disability | Exclusion | Mixed group- and individual-therapy formats | Literature search |
| Godecke et al. | 2013 | Amount of Therapy Matters in Very Early Aphasia Rehabilitation after Stroke: A Clinical Prognostic Model | Exclusion | No randomization | Literature search |
| Goncalves et al. | 2021 | The Benefits of Custom Exergames for Fitness, Balance, and Health-Related Quality of Life: A Randomized Controlled Trial with Community-Dwelling Older Adults | Exclusion | Interventions not comparable between groups | Literature search |
| Hirschmann et al. | 2023 | Group versus individual occupational therapy for toddlers with autism as a means to improve access to public healthcare services. Randomised controlled pilot study | Inclusion |  | Literature search |
| Hiyama et al. | 2016 | Effects of Group-Based Exercise on Range of Motion, Muscle Strength, Functional Ability, and Pain During the Acute Phase After Total Knee Arthroplasty: A Controlled Clinical Trial | Exclusion | No randomization | Literature search |
| Hung et al. | 2010 | Effects of group-based versus individual-based exercise training on motor performance in children with developmental coordination disorder: a randomized controlled pilot study | Inclusion |  | Literature search |
| Hurley et al. | 2007 | Clinical effectiveness of a rehabilitation program integrating exercise, self-management, and active coping strategies for chronic knee pain: a cluster randomized trial | Inclusion |  | Review |
| Iwanaga et al. | 2014 | Pilot Study: Efficacy of Sensory Integration Therapy for Japanese Children with High-Functioning Autism Spectrum Disorder | Exclusion | No randomization | Literature search |
| Jansen et al. | 2018 | Comparison of a group-delivered and individually delivered lifestyle-integrated functional exercise (LiFE) program in older persons: a randomized noninferiority trial | Exclusion | Interventions not comparable between groups | Literature search |
| Janssen et al. | 2001 | The effects of physiotherapy for female urinary incontinence: individual compared with group treatment | Exclusion | Interventions not comparable between groups | Literature search |
| Jones et al. | 2015 | Is group pelvic floor retraining as effective as individual treatment? | Exclusion | No randomization | Literature search |
| Kang et al. | 2020 | Group format rehabilitation is equally effective as individual therapy in patients with surgically repaired rotator cuff tears | Exclusion | No randomization | Literature search |
| Karalı et al. | 2022 | Voice Therapy Efficacy in Pediatric Professional Voice Users With Vocal Fold Nodules: A Preliminary Study | Inclusion |  | Literature search |
| Keenan et al. | 2014 | Effectiveness of Experiential Life Skills Coaching for Youth with a Disability | Exclusion | No randomization | Literature search |
| Ko et al. | 2013 | One-to-One Therapy Is Not Superior to Group or Home-Based Therapy After Total Knee Arthroplasty | Exclusion | Interventions not comparable between groups | Literature search |
| Ko et al. | 2019 | Effect of Group-Task-Oriented Training on Gross and Fine Motor Function, and Activities of Daily Living in Children with Spastic Cerebral Palsy | Exclusion | Interventions not comparable between groups | Literature search |
| Krumov et al. | 2022 | Is group-based physical therapy superior to individual rehabilitation in elderly adults after total knee arthroplasty? A prospective observational study | Exclusion | Interventions not comparable between groups | Literature search |
| Labudek et al. | 2023 | Changes in Psychological Determinants of Behavior Change after Individual versus Group- Based Lifestyle-integrated Fall Prevention: Results from the LiFE-is-LiFE Trial | Exclusion | Interventions not comparable between groups | Literature search |
| Lamb et al. | 2009 | Group treatments for sensitive health care problems: a randomised controlled trial of group versus individual physiotherapy sessions for female urinary incontinence | Exclusion | No randomization | Literature search |
| Lee et al. | 2015 | Individualized feedback-based virtual reality exercise improves older women’s self-perceived health: A randomized controlled trial | Exclusion | Interventions not comparable between groups | Literature search |
| Lee et al. | 2016 | Comparison of individualized virtual reality- and group-based rehabilitation in older adults with chronic stroke in community settings: a pilot randomized controlled trial | Exclusion | Interventions not comparable between groups | Literature search |
| Meerschman et al. | 2019 | Massed Versus Spaced Practice in Vocology: Effect of a Short-Term Intensive Voice Therapy Versus a Long-Term Traditional Voice Therapy | Exclusion | No randomization | Literature search |
| Meerschman et al. | 2020 | The Patient’s Opinion Regarding Different Service Delivery Models for Voice Therapy | Exclusion | No randomization | Literature search |
| Meineche-Schmidt et al. | 2012 | Long-term outcome of multidisciplinary intervention of chronic non-cancer pain patients in a private setting | Exclusion | No randomization | Literature search |
| Miller et al. | 2014 | Mastery motivation as a predictor of occupational performance following upper limb intervention for school-aged children with congenital hemiplegia | Exclusion | Interventions not comparable between groups | Literature search |
| Mitsui et al. | 2021 | Sociability-based fitness approach in Parkinson’s disease: Comparison with conventional rehabilitation | Exclusion | Interventions not comparable between groups | Literature search |
| Mowat et al. | 2022 | Reduced individual treatment delivery has no effect on outcomes in a multidisciplinary pain management program | Exclusion | Mixed group- and individual-therapy formats | Literature search |
| Naumann et al. | 2012 | An individual‐based versus group‐based exercise and counselling intervention for improving quality of life in breast cancer survivors. A feasibility and efficacy study | Exclusion | No randomization | Literature search |
| Nees et al. | 2020 | Multidisciplinary Pain Management of Chronic Back Pain: Helpful Treatments from the Patients’ Perspective | Exclusion | No randomization | Literature search |
| Nerz et al. | 2022 | Group-Based and Individually Delivered LiFE: Content Evaluation and Predictors of Training Response – A Dose-Response Analysis | Exclusion | Interventions not comparable between groups | Literature search |
| Netzer et al. | 2020 | Individual Versus Group Physical Therapy for Patients with Low Back Pain: Outcomes and Process (Therapeutic Factors) | Exclusion | No randomization | Literature search |
| Ohlsson et al. | 2020 | Voice Therapy Outcome—A Randomized Clinical Trial Comparing Individual Voice Therapy, Therapy in Group, and Controls Without Therapy | Exclusion | Interventions not comparable between groups | Literature search |
| O’Keeffe et al. | 2020 | Cognitive functional therapy compared with a group-based exercise and education intervention for chronic low back pain: a multicentre randomised controlled trial (RCT) | Exclusion | Interventions not comparable between groups | Literature search |
| Orui et al. | 2023 | Social Buffering Effects during Craft Activities in Parallel Group Session Revealed by EEG Analysis and Parasympathetic Activity | Exclusion | No group therapy | Literature search |
| Osterloh et al. | 2023 | The effect of a digital‑assisted group rehabilitation on clinical and functional outcomes after total hip and knee arthroplasty—a prospective randomized controlled pilot study | Exclusion | Interventions not comparable between groups | Literature search |
| Pereira et al. | 2011 | Individual and group pelvic floor muscle training versus no treatment in female stress urinary incontinence: a randomized controlled pilot study | Inclusion |  | Literature search |
| Rabiei et al. | 2021 | Comparing Pain Neuroscience Education Followed by Motor Control Exercises With Group-Based Exercises for Chronic Low Back Pain: A Randomized Controlled Trial | Exclusion | Interventions not comparable between groups | Literature search |
| Renner et al. | 2016 | Group therapy task training versus individual task training during inpatient stroke rehabilitation: a randomised controlled trial | Exclusion | Interventions not comparable between groups | Literature search |
| Ricci et al. | 2021 | Acute Cardiometabolic and Perceptual Responses to Individual and Group-Based Body-Weight Resistance Exercise in Girls | Exclusion | Mixed group- and individual-therapy formats | Literature search |
| Sharma et al. | 2024 | Group tele-rehabilitation improves quality of life among subjects with Parkinson’s disease: A two arm non-parallel non-randomized clinical trial | Exclusion | No randomization | Literature search |
| Song et al. | 2015 | Effect of the class and individual applications of task-oriented circuit training on gait ability in patients with chronic stroke | Inclusion |  | Review |
| Soni et al. | 2013 | Randomized Controlled trial of Group Versus Individual Physiotherapy Sessions for Genuine Stress Incontinence in Women | Exclusion | Interventions not comparable between groups | Review |
| Streicher et al. | 2014 | Comparison of group motor control training versus individual training for people suffering from back pain | Exclusion | No randomization | Literature search |
| Surprenant et al. | 2014 | Adapting to Higher Demands: Using Innovative Methods to Treat Infants Presenting With Torticollis and Plagiocephaly | Exclusion | No randomization | Literature search |
| Thieme et al. | 2012 | Mirror therapy for patients with severe arm paresis after stroke – a randomized controlled trial | Inclusion |  | Literature search |
| Toledano-González et al. | 2018 | Occupational Therapy, Self-Efficacy, Well-Being in Older Adults Living in Residential Care Facilities: A Randomized Clinical Trial | Exclusion | Interventions not comparable between groups | Literature search |
| Toledano-González et al. | 2019 | Well-Being, Self-Efficacy and Independence in older adults: A Randomized Trial of Occupational Therapy | Exclusion | Interventions not comparable between groups | Literature search |
| Vadstrup et al. | 2011 | Health-related quality of life and self-related health in patients with type 2 diabetes: Effects of group-based rehabilitation versus individual counselling | Exclusion | Interventions not comparable between groups | Literature search |
| Wuennemann et al. | 2020 | Dose and staffing comparison study of upper limb device-assisted therapy | Exclusion | No randomization | Review |
| Zamani et al. | 2018 | The effects of narrative-based language intervention (NBLI) on spoken narrative structures in Persian-speaking cochlear implanted children: A prospective randomized control trial | Inclusion |  | Literature search |
| Zingmark et al. | 2016 | Occupation-focused health promotion for well older people — A cost-effectiveness analysis | Exclusion | Interventions not comparable between groups | Literature search |

Supplementary Table S5. Summary of included studies

| **Author (year), country** | **Setting** | **Target population** | **Sample size individual therapy** | **Sample size group therapy (group size)** | **Frequency and duration** | **Intervention (in-person or digital)** | **Data collection timepoints** |
| --- | --- | --- | --- | --- | --- | --- | --- |
| **Physiotherapy** | | | | | | | |
| Ciortea et al. (2020), Romania (28) | Clinical rehabilitation hospital | Patients with Parkinson’s disease | 30 | 30  (6) | 10 sessions, each 90 minutes, within 2 weeks | Physical therapy (in-person) | Pretest, posttest |
| Dorrell et al. (2020), United Kingdom (29) | No information | Resistance trained males | 19  (Not reported) | | 2 times a week, within 6 weeks | Strength and power exercises (load-velocity profiling) (in-person) | At least 96 hours before or after the most recent training session |
| Doussoulin et al. (2018), Chile (30) | Community rehabilitation Centre | Patients affected by a stroke | 12 | 24  (4) | 6 sessions, each 180 minutes, within 10 days | Constraint-induced movement therapy (in-person) | Pretest, posttest, follow-up after 6 months |
| Hung et al. (2010), China (32) | Pediatric outpatient physiotherapy department of a hospital | Children with developmental coordination disorder | 11 | 12  (4-6) | 8 sessions, each 45 minutes, once a week, within 8 weeks | Motor skill training (in-person) | Pretest, posttest |
| Hurley et al. (2007), United Kingdom (37) | Community hospital and outpatient physiotherapy department | Patients with chronic knee pain | 146 | 132  (8) | 12 sessions, twice a week, within 6 weeks | Physiotherapy exercises (in-person) | Pretest, posttest, follow-up after 6 months |
| Pereira et al. (2011), Brazil (34) | University research laboratory | Females with stress urinary incontinence | 17 | 17  (8-10) | 12 sessions, each 60 minutes, twice a week, within 6 weeks | Pelvic floor muscle training (in-person) | Pretest, posttest |
| Song et al. (2015), Korea (38) | Hospital | Patients with chronic stroke | 10 | 10  (4-6) | Each session 30 minutes, 3 times a week, within 4 weeks | Task-oriented circuit training (in-person) | Pretest, posttest |
| Thieme et al. (2012), Germany (35) | Inpatient rehabilitation centre | Patients with severe arm paresis after stroke | 18 | 21  (2-6) | 20 sessions, each max. 30 minutes, within 5 weeks | Mirror therapy (in-person) | Pretest, posttest |
| **Speech therapy** | | | | | | | |
| Karalı et al. (2022), Turkey (33) | School | Male students with vocal fold nodules | 8 | 8  (4) | Each session 30 minutes, within 6 weeks | Vocal hygiene therapy and vocal function exercises (in-person) | Pretest, posttest |
| Zamani et al. (2018), Iran (36) | Cochlear implantation and auditory-verbal rehabilitation centers | Cochlear implanted children | 12 | 12  (12) | 24 sessions, each 60 minutes, within 8 weeks | Narrative-based language intervention (in-person) | Pretest, posttest, follow-up after 2 months |
| **Occupational therapy** | | | | | | | |
| Hirschmann et al. (2023), Israel (31) | Pediatric rehabilitation center | Toddlers with autism | 10 | 14  (2-4) | 12 sessions, each 45 minutes, once a week, within 3 months | Pre-planned, semi-spontaneous play sessions (developmental, individual-differences, and relationship-based floortime model) (in-person) | Pretest, posttest |

Supplementary Table S6. Data collection instruments and comparisons of outcomes per study

| **Author** | **Outcome** | **Data collection (instruments)** | **Individual therapy (mean, posttest)** | **Group therapy (mean, posttest)** | **p-value** | **Comparison between individual and group therapy** |
| --- | --- | --- | --- | --- | --- | --- |
| Ciortea et al. (28) | Gait speed | 6-minute walk test | 0.72 | 0.85 | < 0.01 | Favors group therapy |
|  | Gait speed | 10-meter walk test (normal speed) | 0.97 | 1.06 | NS^1^ | No difference |
|  | Gait speed | 10-meter walk test (maximum speed) | 1.18 | 1.21 | NS | No difference |
| Dorrell et al. (29) | Strength’s performance | Back squat one-repetition maximum | 164.8 | 161.4 | NS | No difference |
|  | Jump performance | Countermovement | 41.2 | 37.8 | NS | No difference |
|  |  | Static-squat | 38.1 | 34.2 | NS | No difference |
|  |  | Standing-broad jumps | 103.7 | 90.7 | NS | No difference |
| Doussoulin et al. (30) | Amount of use/ quality of movement | Chilean versions of the Motor Activity Log-30 | 2.69 | 3.09 | < 0.001 | Favors group therapy |
|  | Motor function | Action Research Arm Test | 40.19 | 42.91 | < 0.05 | Favors group therapy |
| Hirschmann et al. (31) | Total score composite | Adaptive Behavior Assessment System questionnaire | 67.6 | 73.7 | NS | No difference |
|  | Concept composite |  | 72.5 | 78 | NS | No difference |
|  | Social composite |  | 64.7 | 76.1 | NS | No difference |
|  | Practical composite |  | 69.9 | 74.7 | NS | No difference |
|  | Fine motor skills | Peabody Developmental Motor Scales Second Edition | 73.5 | 82.3 | NS | No difference |
|  | Quality of life | Pediatric Quality of Life Inventory | 60.4 | 65 | NS | No difference |
|  | Staff burden of all therapists | Self-reported questionnaire | NR^2^ | NR | NS | No difference |
|  | Waiting days | Number of waiting days | 108.8 | 52.4 | < 0.01 | Favors group therapy |
|  | Nonattendance | Absence from a session | 2 | 3.2 | NS | No difference |
| Hung et al. (32) | Total impairment score | Movement Assessment Battery for Children | 10 | 10.4 | NS | No difference |
|  | Manual dexterity |  | 3.9 | 4.6 | NS | No difference |
|  | Ball skills |  | 5.2 | 5 | NS | No difference |
|  | Static/dynamic balance |  | 10 | 10.4 | NS | No difference |
|  | Parental satisfaction | Parental satisfaction questionnaire | 89.5 % | 84.5 % | NS | No difference |
|  | Home exercise compliance | Logbook | 62.2 % | 50.1 % | NS | No difference |
| Hurley et al. (37) | Physical function | Likert version of the Western Ontario and McMaster Universities Osteoarthritis Index | NR | NR | NS | No difference |
|  |  | Quadriceps maximum voluntary contraction | NR | NR | NS | No difference |
|  |  | Quadriceps voluntary activation | NR | NR | NS | No difference |
|  | Objective  functional performance | Aggregate function performance time of four common activities of daily living | NR | NR | NS | No difference |
|  | Pain | Likert version of the Western Ontario and McMaster Universities Osteoarthritis Index pain subscale | NR | NR | NS | No difference |
|  | Health-related quality of life | McMaster Toronto Arthritis Questionnaire | NR | NR | NS | No difference |
|  |  | EuroQol | NR | NR | NS | No difference |
|  | Changes in health beliefs and self-efficacy | Exercise Health Beliefs and Self-Efficacy Questionnaire | NR | NR | NS | No difference |
|  | Anxiety and depression | Hospital Anxiety and Depression Scale | NR | NR | NS | No difference |
| Karalı et al. (33) | S/z ratio | /s/ and /z/ phonation | 1.1 | 0.8 | < 0.05 | Favors individual therapy |
|  | Maximum phonation time | /i/ phonation | NR | NR | NS | No difference |
|  | F0 | Recordings | 213.82 | 180.99 | NS | No difference |
|  | Shimmer |  | 11.71 | 12.95 | NS | No difference |
|  | Jitter |  | 0.9 | 0.5 | NS | No difference |
|  | Noise harmonic ratio |  | 0.423 | 0.216 | < 0.05 | Favors group therapy |
|  | Self-perception of voice disorder | Turkish Children's Voice Handicap Index-10 | 8.7 | 8.3 | NS | No difference |
| Pereira et al. (34) | Urinary loss | Pad test | 0.45 | 0.46 | NS | No difference |
|  | Pelvic floor muscle pressure | Perina stim device | 38.53 | 37.13 | NS | No difference |
|  | Pelvic floor muscle strength | Digital palpation | 2.73 | 3.07 | NS | No difference |
|  | General health | King's Health Questionnaire | 30 | 30 | NS | No difference |
|  | Incontinence impact |  | 17.76 | 28.84 | NS | No difference |
|  | Limitations of daily activities |  | 5.56 | 11.09 | NS | No difference |
|  | Physical limitations |  | 1.11 | 13.31 | NS | No difference |
|  | Social limitations |  | 3.7 | 5.97 | NS | No difference |
|  | Personal relationships |  | 0 | 11.02 | < 0.05 | Favors individual therapy |
|  | Emotions |  | 0 | 11.79 | < 0.05 | Favors individual therapy |
|  | Sleep and disposition |  | 0 | 5.55 | NS | No difference |
|  | Gravity |  | 15.11 | 26.64 | NS | No difference |
|  | Satisfaction with treatment | Questionnaire | 86 % | 86 % | NS | No difference |
| Song et al. (38) | Velocity | GAITRite | 57.5 | 69.2 | NS | No difference |
|  | Cadence |  | 85.6 | 99.9 | NS | No difference |
|  | Stance phase symmetry profile |  | 0.2 | 0.2 | NS | No difference |
|  | Swing phase symmetry profile |  | 0.6 | 0.5 | NS | No difference |
|  | Step length symmetry profile |  | 0.3 | 0.2 | NS | No difference |
|  | Two-minute  walking test |  | 64 | 73.9 | NS | No difference |
| Thieme et al. (35) | Arm function on an impairment level | Fugl-Meyer Test | 8.5 | 8.2 | NS | No difference |
|  | Somatosensory |  | 9.1 | 9.1 | NS | No difference |
|  | Range of motion sections |  | 20.1 | 20.1 | NS | No difference |
|  | Arm function on a functional level | Action Research Arm Test | 5.1 | 1.3 | NS | No difference |
|  | Changes in resistance to passive movement of finger flexor | Ashworth Scale | 3 | 1 | < 0.05 | Favors individual therapy |
|  | Changes in resistance to passive movement of wrist flexor |  | 2 | 2 | NS | No difference |
|  | Visuospatial neglect | Star Cancellation Test | 46 | 43.4 | NS | No difference |
|  | Basic activities of daily living | Barthel Index | 57.2 | 56.8 | NS | No difference |
|  | Pain | Fugl-Meyer Test | 18.3 | 19.6 | NS | No difference |
|  | Quality of life | Stroke Impact Scale | 56.3 | 55.3 | NS | No difference |
| Zamani et al. (36) | Productivity of spoken narrative | Number of total words | 30.5 | 30.5 | NS | No difference |
|  |  | Number of cohesive markers | 5 | 5 | NS | No difference |
|  |  | Number of total clauses | 11.5 | 10.5 | NS | No difference |
|  | Complexity of spoken narrative | Average T-unit length | 3.5 | 3.4 | NS | No difference |
|  |  | Subordination Index | 1.39 | 1.44 | NS | No difference |
|  | Accuracy of spoken narrative | Semantic/syntactic accuracy | 72.8 % | 70.3 % | NS | No difference |
|  | Story grammar of spoken narrative | Number of story grammars | 6 | 7 | NS | No difference |
| ^1^NS: Not significant.  ^2^NR: Not reported.  ^3^The results for the comparison between individual and group therapy were reported not post-therapy but rather at a follow-up conducted six months later. | | | | | | |
